# Chemical Analysis of Morphological Changes in Lysophosphatidic Acid-Treated Ovarian Cancer Cells

**DOI:** 10.1038/s41598-017-15547-7

**Published:** 2017-11-10

**Authors:** Karen A. Bailey, Yuliya Klymenko, Peter E. Feist, Amanda B. Hummon, M. Sharon Stack, Zachary D. Schultz

**Affiliations:** 1 Department of Chemistry and Biochemistry, University of Notre Dame, Notre Dame, IN 46556 USA; 2 Harper Cancer Research Institute, University of Notre Dame, South Bend, IN 46617 USA

## Abstract

Ovarian cancer (OvCa) cells are reported to undergo biochemical changes at the cell surface in response to treatment with lysophosphatidic acid (LPA). Here we use scanning electron microscopy (SEM) and multiplex coherent anti-Stokes Raman scattering (CARS) imaging via supercontinuum excitation to probe morphological changes that result from LPA treatment. SEM images show distinct shedding of microvilli-like features upon treatment with LPA. Analysis of multiplex CARS images can distinguish between molecular components, such as lipids and proteins. Our results indicate that OvCa429 and SKOV3ip epithelial ovarian cancer cells undergo similar morphological and chemical responses to treatment with LPA. The microvilli-like structures on the surface of multicellular aggregates (MCAs) are removed by treatment with LPA. The CARS analysis shows a distinct decrease in protein and increase in lipid composition on the surface of LPA-treated cells. Importantly, the CARS signals from cellular sheddings from MCAs with LPA treatment are consistent with cleavage of proteins originally present. Mass spectrometry on the cellular sheddings show that a large number of proteins, both membrane and intracellular, are present. An increased number of peptides are detected for the mesenchymal cell line relative to the epithelial cell indicating a differential response to LPA treatment with cancer progression.

## Introduction

Lysophosphatidic acid (LPA) plays a key role in numerous biological processes, such as the stimulation of cell migration, invasion, and cancer metastasis^1–5^. Found at increased concentrations as high as 80 μM in the ascites fluid of the peritoneal cavity in ovarian cancer patients, LPA is a putative biomarker for the disease^6^. LPA is reported to interact through multiple pathways, often with redundant mechanisms, resulting in altered expression of many proteins that have made it difficult to clarify the role of this molecule in ovarian cancer and other diseases. At these elevated concentrations, LPA is reported to have significant effects on the morphology of cells^7,8^, as well as interfering with the junctions between cells^9–13^. Microscopy techniques that can resolve biochemical changes in cells in response to LPA treatment may help elucidate the activity of this cancer biomarker.

Coherent anti-Stokes Raman scattering (CARS) is a powerful tool for biomedical analysis that provides label-free morphological and chemical contrast with video-rate imaging comparable to fluorescence microscopy without the use of exogenous agents^14–17^. Single-band CARS imaging is most commonly used to probe CH_2_ oscillators in biological systems. By plotting the CARS intensity as a function of coordinate, a high contrast image generally outlines the lipid membrane, fatty acids, and nucleus of the cells in a given tissue sample. This property has been helpful in diagnosing a variety of carcinomas originating from lung, breast, skin, brain, and prostate cancer^18–24^.

In single-band CARS, a pump beam $({{\rm{\omega }}}_{{\rm{P}}})$, Stokes beam $({{\rm{\omega }}}_{{\rm{S}}})$, and probe beam $({{\rm{\omega }}}_{{\rm{P}}^{\prime} }$; typically, ${{\rm{\omega }}}_{{\rm{P}}^{\prime} }={{\rm{\omega }}}_{{\rm{P}}})$ interact with a sample in a four wave mixing process. When the pump and Stokes beams are spatially and temporally overlapped with a beat frequency resonant with a vibrational band, ${{\rm{\omega }}}_{{\rm{P}}}-{{\rm{\omega }}}_{{\rm{S}}}={{\rm{\Omega }}}_{{\rm{vib}}}$, a strong, directional anti-Stokes signal is generated at ${{\rm{\omega }}}_{{\rm{AS}}}=2{{\rm{\omega }}}_{{\rm{P}}}-{{\rm{\omega }}}_{{\rm{S}}}$. While single-band CARS provides rapid imaging capabilities, chemical information present in the Raman spectrum is difficult to discern. An alternative approach is multiplex CARS, where a broadband Stokes beam or supercontinuum excitation is used to probe multiple vibrational bands simultaneously^25–27^. Thus, the multiplex CARS signal is composed of resonant contributions from excited vibrational bands, and nonresonant contributions from the surrounding medium not in tune with the beat frequency. Although the nonresonant background shows complex interference with the resonant signal, there have been advances in instrumentation and data analysis to suppress the nonresonant contributions and reconstruct the spontaneous Raman spectrum from the CARS signal^28–31^.

More recently, multiplex CARS has been implemented as a high spectral resolution imaging technique, in which single-band CARS acquisitions are taken sequentially by manually tuning the Stokes beam, providing more chemical information from the surrounding biomolecules in the focal volume without sacrificing image quality. The reconstructed multiplex CARS spectrum from the high spectral resolution image has the ability to distinguish between lipids and proteins for clinical diagnostics^32^. This multiplex CARS analysis is capable of identifying and delineating different brain tumors based on the varying lipid and protein distributions^33^.

In this report, we utilize the combination of scanning electron microscopy (SEM), multiplex coherent anti-Stokes Raman scattering imaging, and mass spectrometry to investigate the biochemical signals associated with morphology changes on the surface of two distinct ovarian cancer lines resulting from treatment with LPA.

## Results

**Figure 1:**
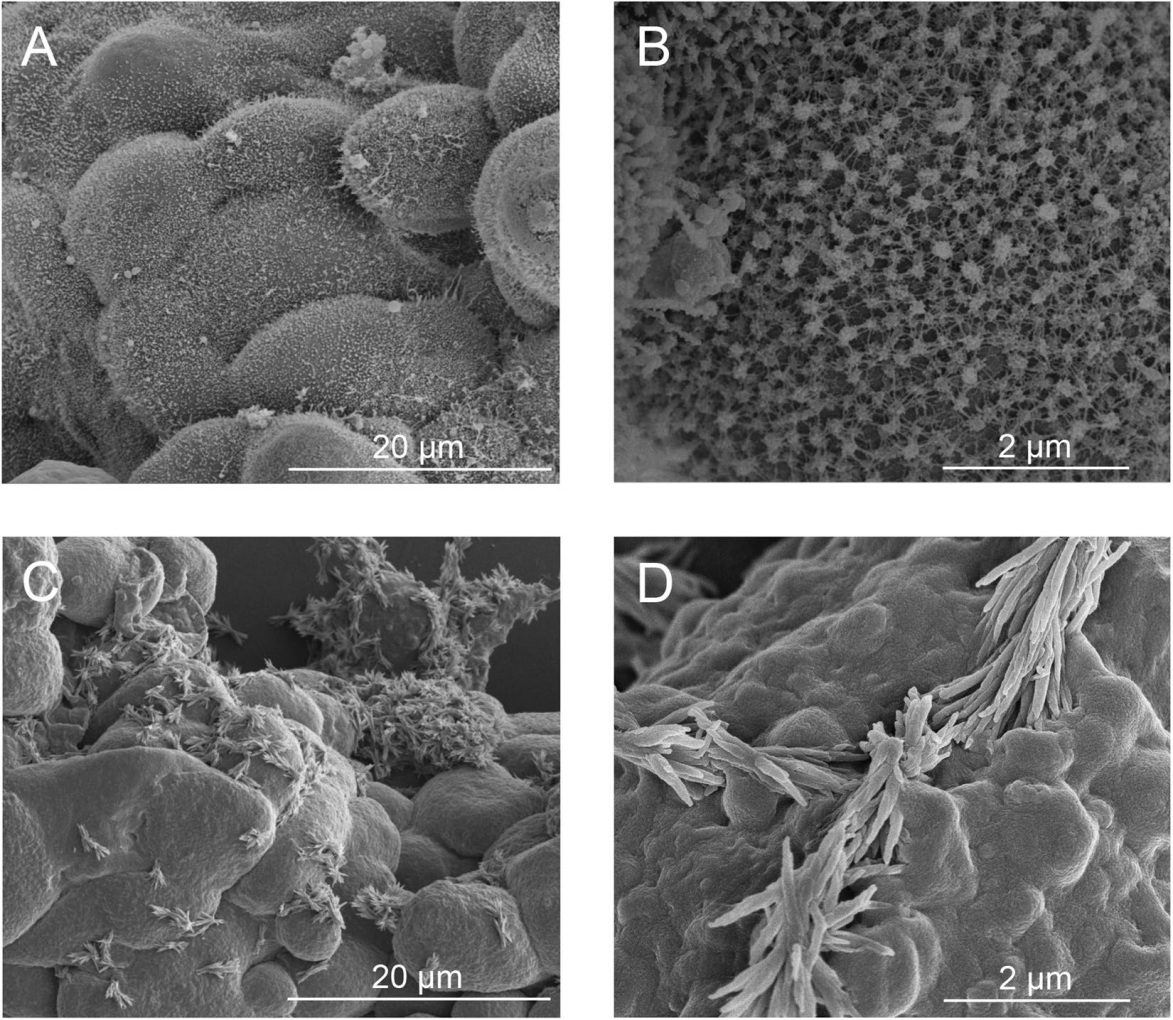
Representative (**A**,**C**) low and (**B**,**D**) high magnification SEM images of (top) non-treated OvCa429 and (bottom) LPA-treated OvCa429 MCAs, respectively.

Observed on most cell surfaces, microvilli are slender extensions of the cell membrane structured with a central core of parallel actin filaments compact with cross-linking actin-binding proteins^34–36^. Specifically for epithelial ovarian cancer cells, there is an abundance of microvilli that appear as dense microscopic protrusions on the cell surface^37–39^. Fig. 1A,B show SEM images of non-treated OvCa429 multicellular aggregates with a dense uniform coverage of microvilli. However, the addition of 80 µM lysophosphatidic acid in the culture medium induces morphological changes displayed in the SEM images of the LPA-treated OvCa429 multicellular aggregates in Fig. 1C,D. The LPA-treated OvCa429 MCAs have a bare, smooth cell surface that suggest the loss or reconstruction of microvilli and cell-surface proteins correspond to the cellular sheddings observed and newly formed larger protrusions. Given the many biological roles associated with LPA, these changes are not surprising. In ovarian cancer, LPA is reported to promote E-cadherin ectodomain shedding in OvCa429 cells^5^. Ovarian cancer has also been linked to increased concentrations of exosomes in ascites fluid^40^. The biological mechanism that causes the physical changes observed in Fig. 1C,D is unclear.

Multiplex CARS imaging can provide insight to the localization and chemical composition of these samples by detecting the intrinsic vibrational signal of biomolecules on the cell surface. A recent study reported the advantages of analyzing the C-H region from −2800 to −3000 cm^−1^, in which the distinct ratio of methylene, CH_2_, to methyl, CH_3_, groups can differentiate molecular markers such as lipids and proteins^32^. The peak ratio between CH_2_/CH_3_ functional groups is high in lipids, on average >10, consistent with literature. The peak ratio is on average <1 for proteins. In their report, Meyer *et al*. observed the spectral resolution of the measurement was determined by the fiber-based excitation lasers with a spectral width of 0.8 cm^−1^ ^32^. In our instrument, the spectral resolution is time-bandwidth product limited to 7 cm^−1^; however, sufficient resolution remains to apply the analysis method reported by Meyer *et al*. The reconstructed multiplex CARS spectrum is approximated by the imaginary term of the third-order susceptibility, ${\chi }_{Ri}$, which corresponds to the Raman spectrum for signals that have a high ratio of CARS intensity, A, to the magnitude of the nonresonant background.

**Figure 2:**
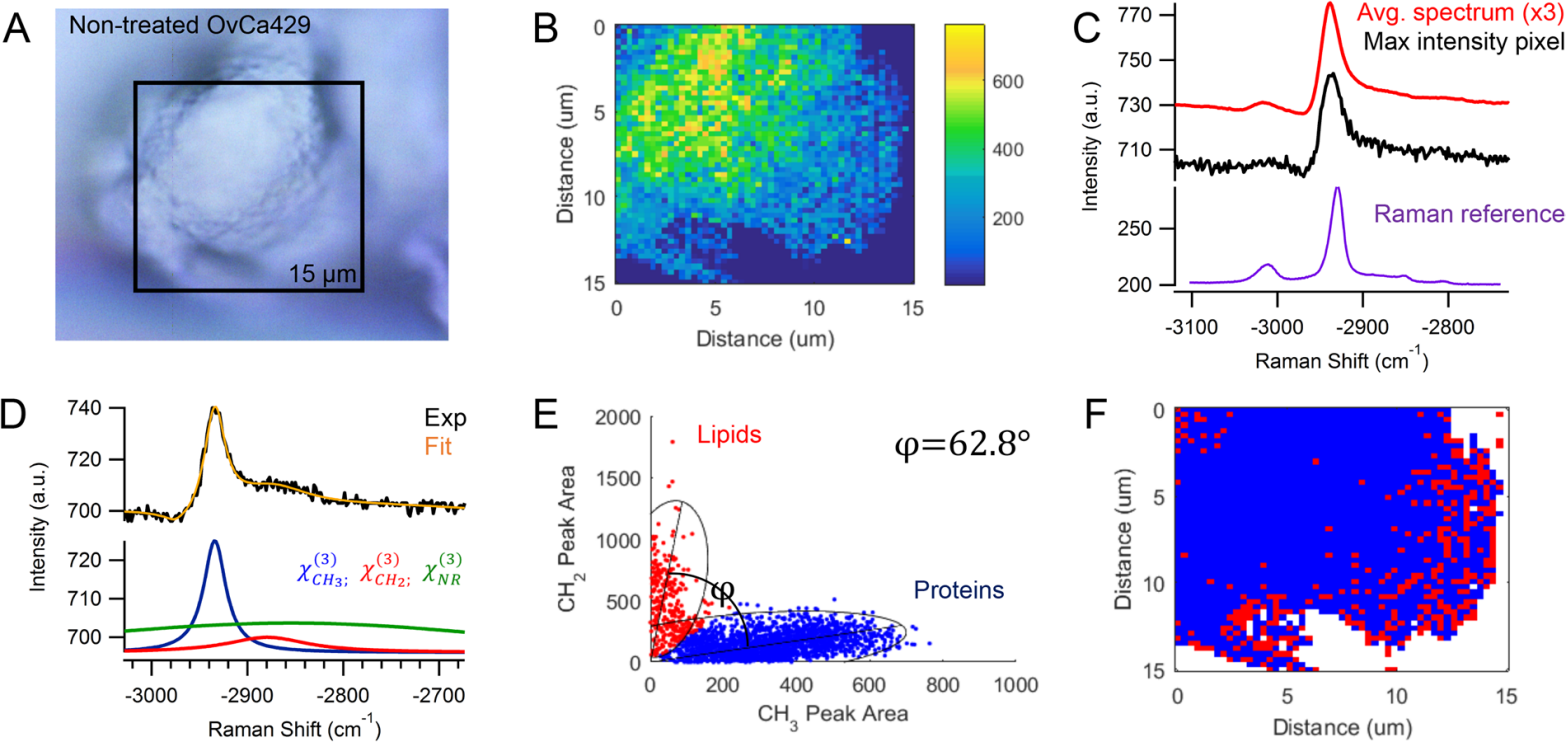
(**A**) Brightfield image focused on non-treated OvCa429 cells on the surface of the multicellular aggregate is in good agreement with (**B**) CARS image of −2930 cm^−1^ peak area. The comparison of (**C**) the maximum intensity CARS spectrum and average spectrum of the map show similar spectral structure across the cell surface of the sample. The spontaneous Raman spectrum obtained from OvCa429 cells is plotted over the equivalent Raman shifts for comparison (See Fig. S3 for more details). (**D**) The CARS intensity (black) is fit (orange) to Eq. 1 to ascertain the nonresonant background (green) and two resonant contributions from the CH_2_ and CH_3_ vibrational bands at −2850 cm^−1^ (red) and −2930 cm^−1^ (blue), respectively. (**E**) The frequency scatter plot distinguishes two spectral clusters with 95% confidence ellipses indicative of lipids (red) and proteins (blue). The spectral separation is described by the separation angle, φ, from the ratios of CH_2_/CH_3_ signal. (**F**) The reconstructed map shows the chemical distribution of the segmented lipid (red) and protein (blue) pixels. Experimental parameters: Pump = 720 nm, 26.50 mW; Supercontinuum = 785–945 nm, 3.70 mW; Acquisition time = 500 ms/pixel; Map size = 51 × 51 pixels; Steps = 300 nm; Objective = 100x, 0.9 NA.

Figure 2 shows the results of colocalization analysis applied to OvCa429 samples. The CARS image in Fig. 2B correlates well with the brightfield image of a single OvCa429 cancer cell in a multicellular aggregate in Fig. 2A. Depicted in Fig. 2C, the multiplex CARS spectrum of the maximum intensity pixel is an example of the CARS signal detected across the cell surface, which is verified by comparison with the average spectrum of the map. There is a strong peak at −2930 cm^−1^ with a shoulder at −2850 cm^−1^ indicative of CH_3_ and CH_2_ vibrational bands, respectively. There is also a weak peak at −3015 cm^−1^ from a CH vibrational band near a C = C bond, likely associated with unsaturated lipids. We describe the multiplex CARS intensity, ${{\rm{I}}}_{{\rm{CARS}}}$, from −2800 to −3000 cm^−1^ as:

1 $${{\rm{I}}}_{{\rm{CARS}}}\,{\rm{\alpha }}{|{{\rm{\chi }}}_{{\rm{NR}}}^{(3)}+\frac{{{\rm{A}}}_{{\rm{R}}1}}{({{\rm{\omega }}}_{{\rm{P}}}-{{\rm{\omega }}}_{{\rm{S}}}-{{\rm{\Omega }}}_{{\rm{R}}1})-{{\rm{i}}{\rm{\Gamma }}}_{{\rm{R}}1}}+\frac{{{\rm{A}}}_{{\rm{R}}2}}{({{\rm{\omega }}}_{{\rm{P}}}-{{\rm{\omega }}}_{{\rm{S}}}-{{\rm{\Omega }}}_{{\rm{R}}2})-{{\rm{i}}{\rm{\Gamma }}}_{{\rm{R}}2}}|}^{2}$$

where each resonant term at −2930 cm^−1^ and −2850 cm^−1^ is characterized by a Lorentz line shape with amplitude, ${\rm{A}}$, bandwidth, ${\rm{\Gamma }}$, and center frequency, Ω^32,41^. The nonresonant background,${\,{\rm{\chi }}}_{{\rm{NR}}}^{(3)}$, is expressed as a complex Gaussian function to account for any coherent interference and contributions from the broadband supercontinuum excitation and surrounding medium:

2 $${{\rm{\chi }}}_{{\rm{NR}}}^{(3)}={{\rm{A}}}_{{\rm{NR}}}{{\rm{e}}}^{{\rm{it}}-(\frac{{({{\rm{\omega }}}_{{\rm{P}}}-{{\rm{\omega }}}_{{\rm{S}}}-{{\rm{\omega }}}_{{\rm{NR}}})}^{2}}{2{{\rm{\Gamma }}}_{{\rm{NR}}}^{2}})}$$

The CARS intensity and spectral fitted line show good agreement in Fig. 2D. The coherent mixing of nonresonant and resonant terms produces the asymmetrical line shape and negative dip on the blue end of the detected signal. (For more details on the nonlinear peak fit functions, see supporting information).

The ratio of the CH_2_ vs. CH_3_ peak intensity observed in CARS spectra can determine whether lipids or proteins predominantly contribute to the methylene stretching region^32^. In Meyer *et al*.’s previous report, a three-dimensional frequency scatter plot was constructed, corresponding to the intensity values of the CH_2_ and CH_3_ signal detected by two image channels. Biomolecules of different intensity ratios at two different frequencies accumulate at different areas of the plot, such that the segments are separated by an angle, φ. This separation angle describes how well the frequency plot can distinguish between two different molecular markers. Since each spectrum contains contributions from lipids, proteins, and potentially other molecules, changes in the relative biochemical composition can alter the separation angle. A poor separation angle may arise from two different frequencies too close together that may be difficult to spectrally resolve, or from a biochemical composition that does not favor one component. Two distinct CARS intensities with minimal variations may result in only one intensity segment in the frequency plot, such that the separation angle is 0, and the two biomolecules are indiscernible.

The multiplex CARS measurements shown have sufficient spectral resolution that the fit functions clearly discern the resonant contribution of the methyl and methylene signals. The peak area of each resonant contribution is plotted as a two-dimensional histogram in Fig. 2E, such that the frequency scatter plot exhibits two spectral clusters. Clusters with higher CH_3_ content are associated with proteins, while higher CH_2_ content is characteristic of lipids. The separation angle, φ, between the two clusters describes how well the 2D-frequency plot can distinguish between two distinct functional groups. The segmented spectral pixels in Fig. 2E can be reconstructed into a map, in Fig. 2F, that illustrates the chemical distribution of predominantly lipid and protein regions complimentary to the CARS image in Fig. 2B.

**Figure 3:**
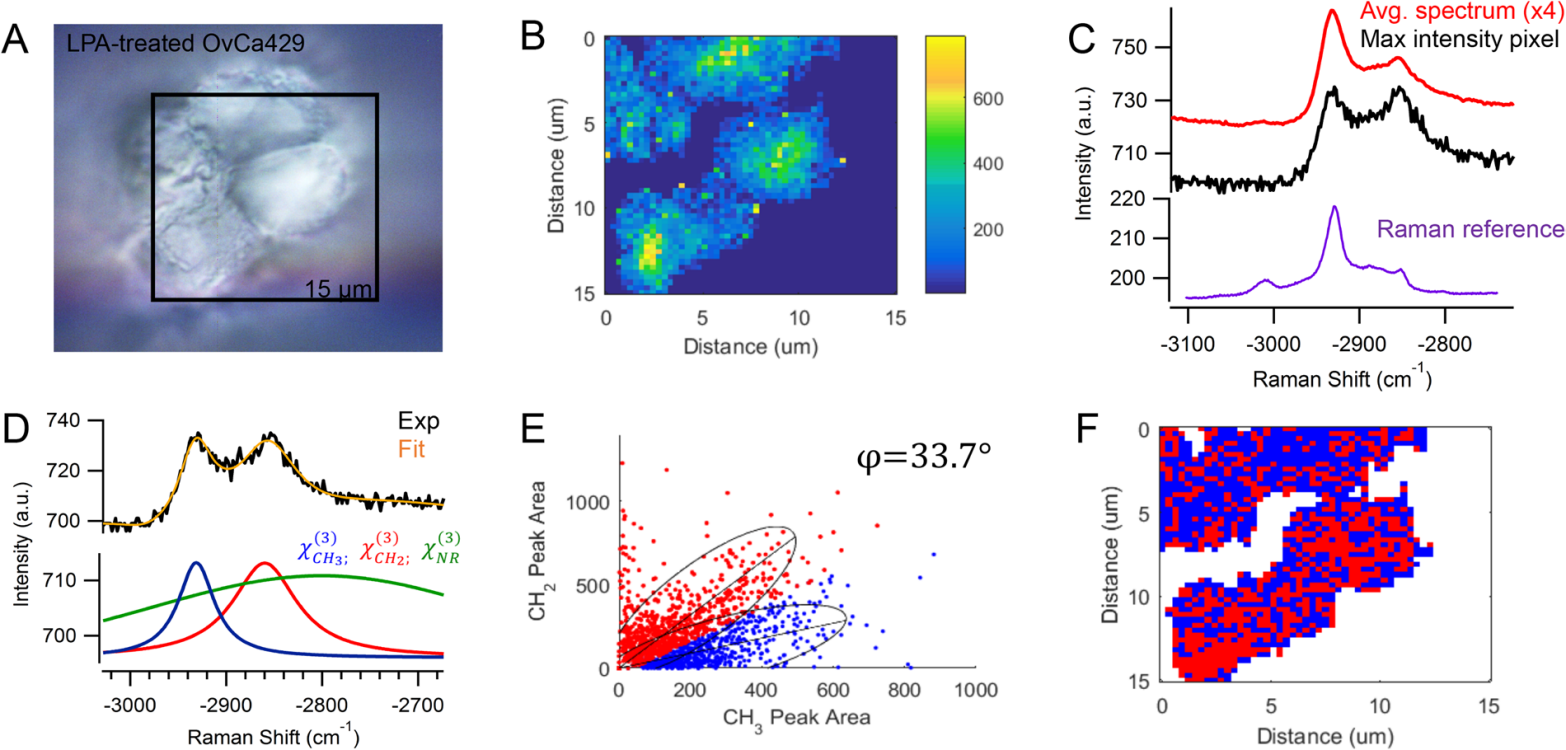
(**A**) Brightfield image of OvCa429 cells on the surface of MCA treated with LPA correlates with (**B**) the CARS image of −2930 cm^−1^ peak area. (**C**) The maximum intensity CARS spectrum and average spectrum of the map show varying contributions of CH_2_ and CH_3_ signal. The spontaneous Raman spectrum obtained from LPA treated OvCa429 cells is plotted over the equivalent Raman shifts for comparison (See Fig. S3 for more details). (**D**) The nonlinear peak fit (orange) of the CARS intensity (black) is composed of nonresonant background (green) and resonant contributions from −2850 cm^−1^ (red) and −2930 cm^−1^ (blue) vibrational bands, respectively. (**E**) The frequency scatter plot displays overlapping spectral clusters with 95% confidence ellipses and angle, φ, corresponding to the spectral separation of lipids (red) and proteins (blue). (**F**) The reconstructed map shows the chemical distribution of the segmented lipid (red) and protein (blue) pixels. Experimental parameters: Pump = 720 nm, 26.50 mW; Supercontinuum = 785–945 nm, 3.70 mW; Acquisition time = 500 ms/pixel; Map size = 51 × 51 pixels; Steps = 300 nm; Objective = 100x, 0.9 NA.

In Fig. 3, LPA-treated OvCa429 MCAs are analyzed in the same manner. The brightfield image of several LPA-treated OvCa429 cells in Fig. 3A and CARS image in Fig. 3B are in agreement with respect to the integrated −2930 cm^−1^ signal. By comparing the multiplex CARS spectra in Fig. 3C, there is a clear increase in −2850 cm^−1^ signal and decrease of −3015 cm^−1^ signal. The resonant contributions of the CH_2_ and CH_3_ stretches are again determined by the spectral fit in Fig. 3D, and plotted as a function of frequency and peak area in Fig. 3E. The separation angle is much lower, suggesting a change in the biochemical composition. The peak ratio between CH_2_/CH_3_ functional groups can discern which spectral pixels are comprised of predominantly lipids or proteins. Based on the chemical distribution map in Fig. 3F, there is a mixture of proteins and lipids on the cell surface, with an increase in pixels comprised predominantly of lipids. The optical resolution (approximately 300 nm) is insufficient to directly correlate the chemical observations in the CARS measurement to the changes observed in electron microscopy (Fig. 1C,D); however, it does signify interesting trends associated with LPA treatment.

**Figure 4:**
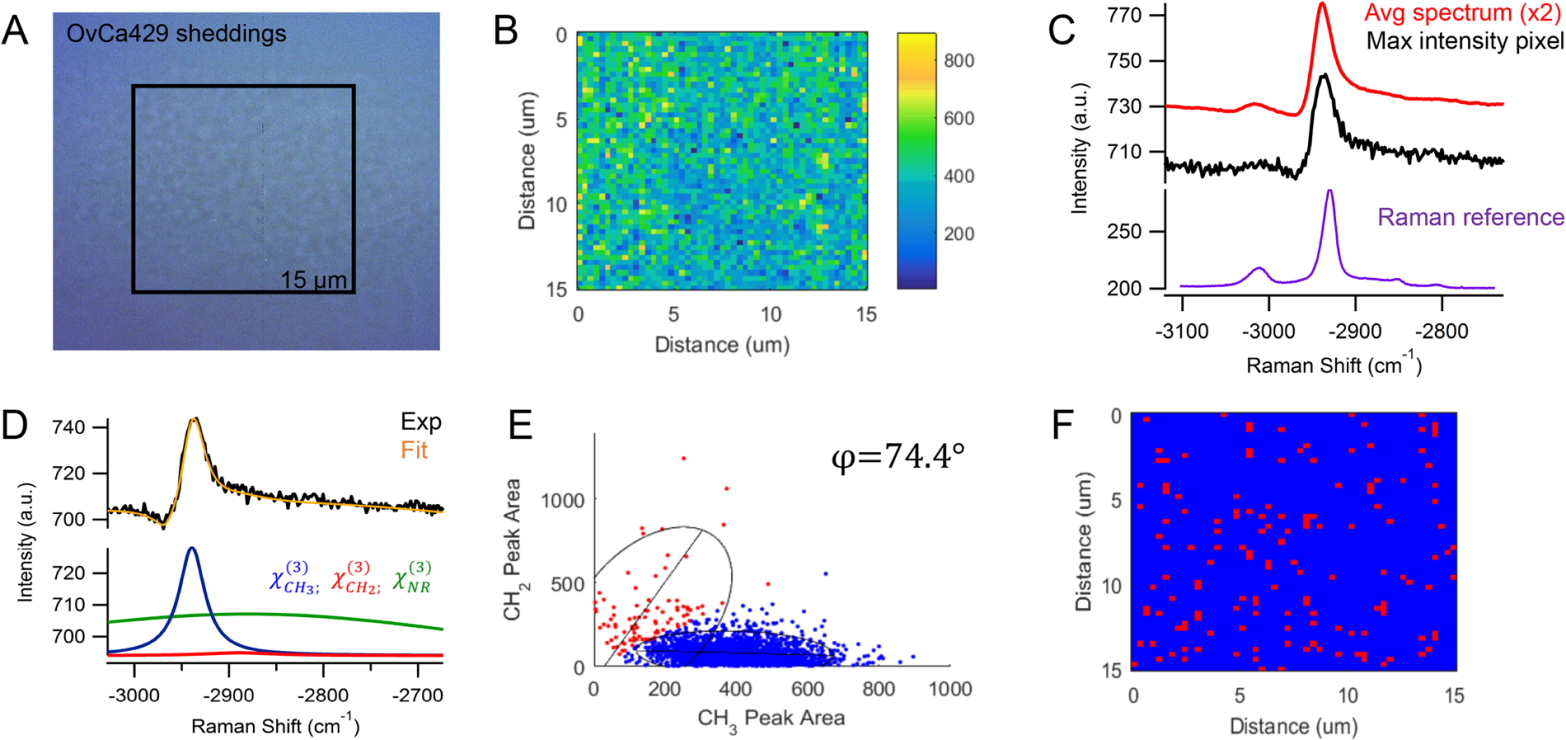
(**A**) Brightfield image of cellular shedding from LPA-treated OvCa429 sample has (**B**) corresponding CARS image of −2930 cm^−1^ peak area. (**C**) The maximum intensity CARS spectrum and average spectrum of the map show similar chemical composition as cell surface of non-treated OvCa429 samples. The spontaneous Raman spectrum obtained from the LPA induced sheddings from OvCa429 cells is plotted over the equivalent Raman shifts for comparison (See Fig. S3 for more details). (**D**) The representative CARS spectrum and nonlinear peak fit results provide the resonant CH_2_ and CH_3_ components used for (**E**) the frequency scatter plot separating the chemical contributions of lipids (red) and proteins (blue). (**F**) The reconstructed map shows the chemical distribution of the segmented lipid (red) and protein (blue) pixels. Experimental parameters: Pump = 720 nm, 26.50 mW; Supercontinuum = 785–945 nm, 3.70 mW; Acquisition time = 500 ms/pixel; Map size = 51 × 51 pixels; Steps = 300 nm; Objective = 100x, 0.9 NA.

To further assess the changes associated with LPA treatment, the cellular sheddings surrounding the LPA-treated OvCa429 MCAs were extracted from the conditioned medium and further characterized in Fig. 4. The brightfield and CARS images of the collected sheddings are shown in Fig. 4A and B, respectively. The multiplex CARS spectra of the cellular sheddings in Fig. 4C have similar intensity ratios for the −2930 and −3015 cm^−1^ vibrational bands and a reduced spectral shoulder at −2850 cm^−1^ when compared to the CARS signal in Fig. 2C. The decrease in lipid contribution is attributed to the isolation of the sheddings, such that the lipid membrane encompassing the MCAs are not present in the focal volume as previously shown in Figs 2 and 3. By evaluating the nonresonant and resonant contributions in Fig. 4D and E, the frequency scatter plot displays an over populated cluster of protein-dominated spectral pixels, emphasized in Fig. 4F. The colocalization analysis indicates the sheddings are essentially composed of proteins.

**Figure 5:**
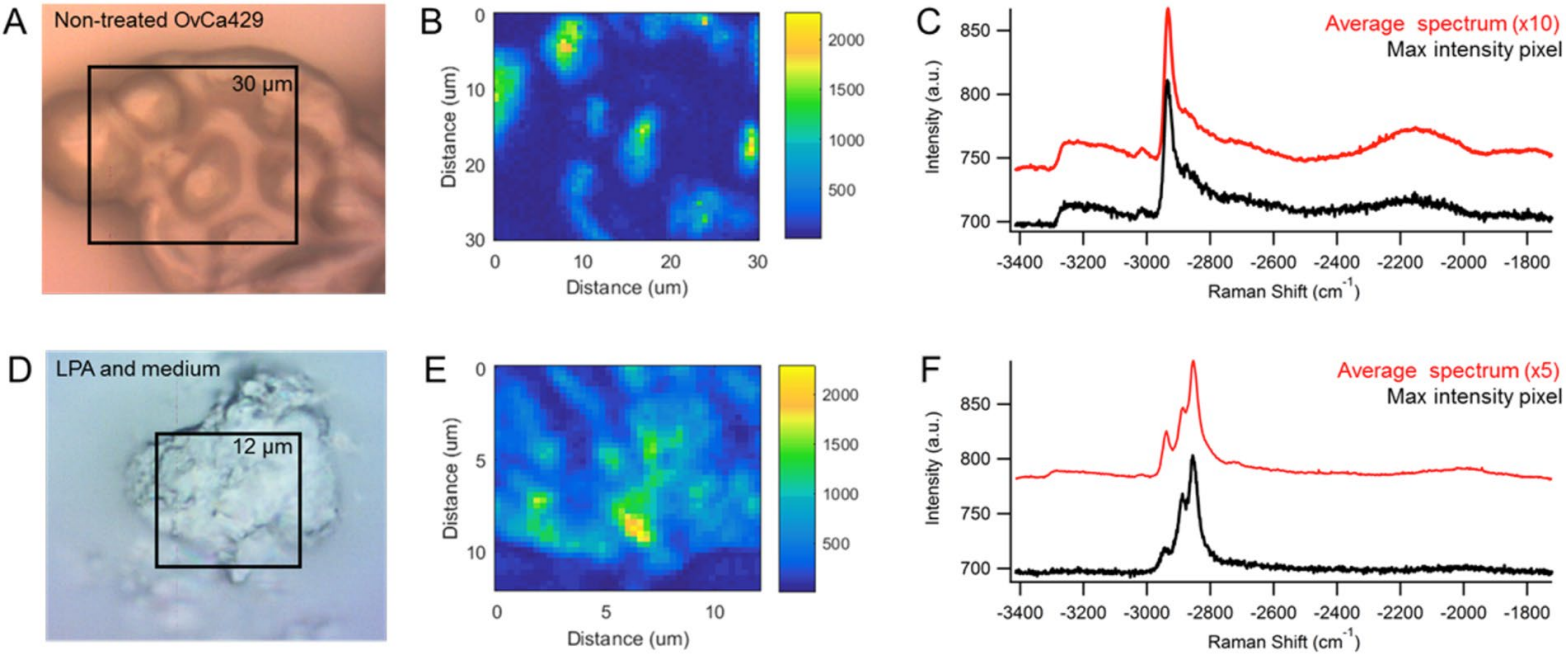
(**A**) Brightfield image of non-treated OvCa429 MCA with (**B**) CARS image of −2930 cm^−1^ has high CH_3_ signal displayed in the (**C**) the maximum intensity pixel and average spectrum. Experimental parameters: Pump = 720 nm, 22.50 mW; Supercontinuum = 785–945 nm, 5.05 mW; Acquisition time = 500 ms/pixel; Map size = 51 × 51 pixels; Steps = 600 nm; Objective = 40x, 0.75NA. (**D**) Brightfield image of crystallized LPA in conditioned medium correlates well with the (**E**) CARS image of −2850 cm^−1^ peak area. (**F**) The CARS spectra from the maximum intensity pixel and map average show three peaks at −2850, −2880, and −2930 cm^−1^ from a combination of symmetric CH_2_, asymmetric CH_2_, and CH_3_ stretches, respectively. Experimental parameters: Pump = 720 nm, 25.20 mW; Supercontinuum = 785–945 nm, 3.80 mW; Acquisition time = 500 ms/pixel; Map size = 41 × 41 pixels; Steps = 300 nm; Objective = 100x, 0.9 NA.

Several control experiments were performed to gain a better understanding of the observed multiplex CARS signal from these OvCa429 samples. In Fig. 5A and B, the brightfield and CARS images of a 30 × 30 µm region contain multiple cells of diverse size and shape within the non-treated OvCa429 MCA. The multiplex CARS spectra in Fig. 5C are consistent throughout the MCA, similar to the CARS spectra detected across a single ovarian cancer cell in Fig. 2C. Additionally, a sample of the culture environment, solely comprised of lysophosphatidic acid and culture medium, is evaluated in Fig. 5D–F. The maximum intensity pixel and average spectrum of the map in Fig. 5F exhibit a strong signal at −2850 cm^−1^ with a shoulder near −2880 cm^−1^ from the symmetric and asymmetric CH_2_ stretches of the phospholipid acyl chain, respectively^26^. The weak signal at −2930 cm^−1^ is indicative of the methyl group at the omega end of LPA. The CARS signal was negligible from the surrounding conditioned medium. Depth profiling (supporting information, Fig. S2) indicates an axial resolution of about 2–3 micrometers, as expected by the coherence lengths in our set-up. This depth resolution may include some of the interior of the cell; however, depth profiling indicates the peak ratios are relatively constant over the focal volume.

Spontaneous Raman imaging was used to verify the chemical trends seen in the three individual OvCa429 samples (supporting information, Fig. S3). The brightfield image, Raman map, and Raman spectra of non-treated OvCa429 MCAs are in agreement with the multiplex CARS analysis in Fig. 2A–C, specifically, the Raman bands at −2930 and −3015 cm^−1^ are observed with both techniques arising from the CH_3_ and CH stretches. The spontaneous Raman spectra provide confidence in the bands used to interpret the nonlinear CARS response. The Raman signal of LPA-treated OvCa429 shows an increased 2850 cm^−1^ intensity similar to the CARS spectra in Fig. 3A–C. However, the −3015 cm^−1^ vibrational band is evident in the Raman spectrum and miniscule in the CARS spectrum. This signal is likely due to the linear relationship between spontaneous Raman intensity and analyte concentration; whereas the CARS intensity is quadratically proportional, such that it becomes more difficult to detect bands present in very low concentrations. As expected, the spontaneous Raman signal of cellular sheddings from LPA-treated OvCa429 cells correlate well with the Raman signal from non-treated OvCa429 cells. The advantage of CARS for this analysis is the significant decrease in time required for each image.

The changes observed with LPA treatment could arise from residual LPA on the cell surface. Indeed, LPA crystals can be observed in brightfield images of MCAs (supporting information, Fig. S4). The characterization of LPA in conditioned medium, depicted in Fig. 5D–F, provides a spectral reference to identify regions of crystallized LPA on the MCAs. The CARS intensity of the −2850 cm^−1^ vibrational band correlates well with the morphological features on the cell surface. However, the spectrum of crystalline LPA is different than the spectrum observed in predominantly lipid areas, enabling differentiation in the molecular origin of the signal.

**Figure 6:**
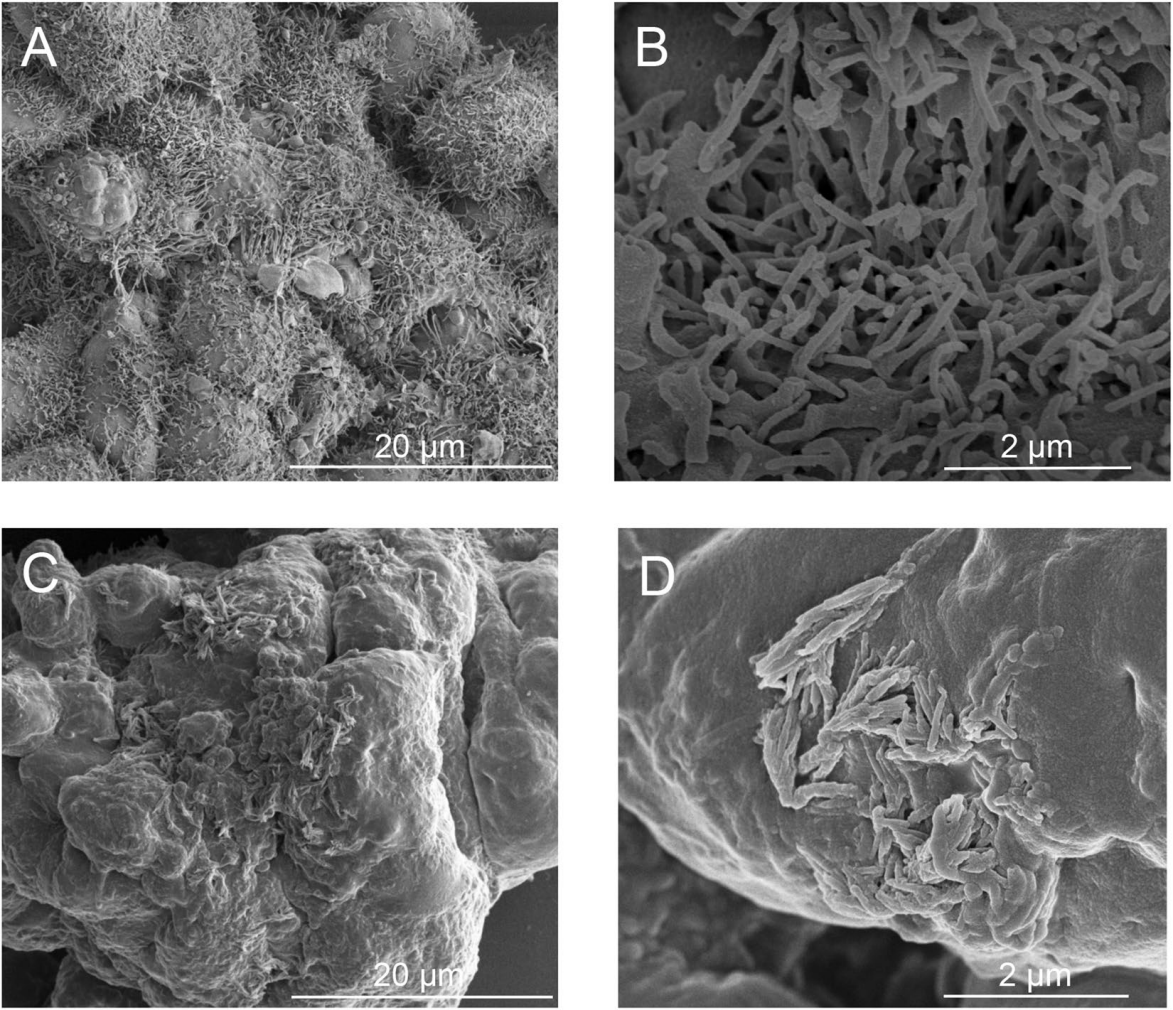
Representative (**A**,**C**) low and (**B**,**D**) high magnification SEM images of (top) non-treated SKOV3ip and (bottom) LPA-treated SKOV3ip MCAs, respectively.

**Figure 7:**
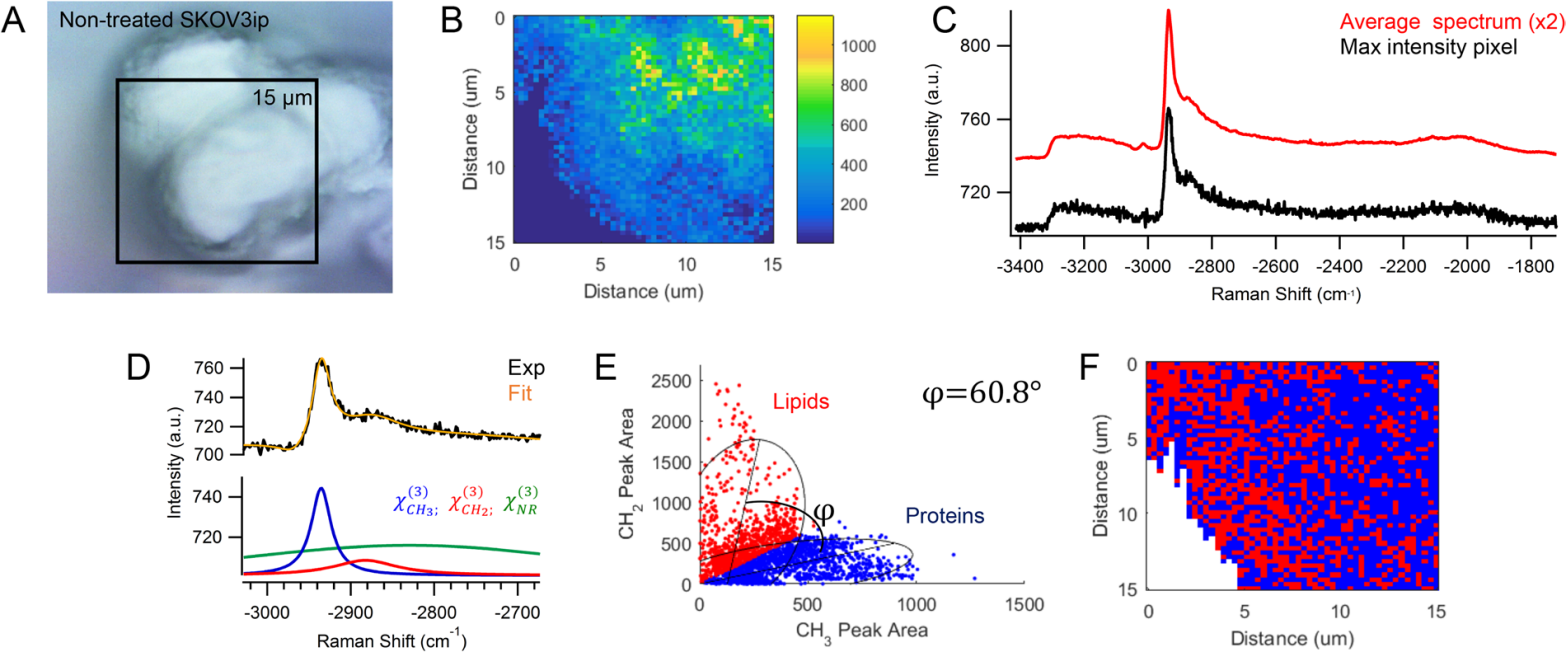
(**A**) Brightfield image focused on non-treated SKOV3ip cells of the MCA correlates well with (**B**) CARS image of −2930 cm^−1^ peak area. The comparison of (**C**) the maximum intensity CARS spectrum and average spectrum of the map show similar chemical composition as non-treated OvCa429 samples. (**D**) The CARS intensity (black) and nonlinear peak fit (orange) is composed of a nonresonant background (green) and two resonant contributions from CH_2_ and CH_3_ bands at −2850 cm^−1^ (red) and −2930 cm^−1^ (blue), respectively. (**E**) Two spectral clusters with 95% confidence ellipses are discernible in the frequency scatter plot corresponding to lipids (red) and proteins (blue) and φ is the separation angle describing the spectral separation of methylene and methyl groups. (**F**) The reconstructed map shows the chemical distribution of the segmented lipid (red) and protein (blue) pixels. Experimental parameters: Pump = 720 nm, 25.20 mW; Supercontinuum = 785–945 nm, 3.80 mW; Acquisition time = 500 ms/pixel; Map size = 51 × 51 pixels; Steps = 300 nm; Objective = 100x, 0.9 NA

A second epithelial ovarian cancer cell line, SKOV3ip, was also examined. SKOV3ip is known to have different cell-surface proteins that are responsible for cell-cell adhesion. Notably, SKOV3ip is a mesenchymal-type cell line that expresses N-cadherin proteins, whereas OvCa429 is an epithelial-type cell line that expresses E-cadherin proteins^38,42^. Interestingly, the SEM images of SKOV3ip MCAs in Fig. 6 undergo similar morphological changes in response to LPA treatment. In Fig. 6A,B, the non-treated SKOV3ip MCAs have abundant microvilli that are longer and thicker in structure, and unevenly formed on the cell surface. The brightfield image of the non-treated SKOV3ip MCA in Fig. 7A is in good agreement with the CARS image in Fig. 7B. In Fig. 7C, the CARS spectra have a more prominent shoulder at −2850 cm^−1^ from the CH_2_ stretches associated with the lipid membrane of the MCA shown in Fig. 6B. Based on the nonlinear spectral fit in Fig. 7D and frequency scatter plot in Fig. 7E, the spectral clusters indicative of lipids and proteins have an equivalent spectral separation angle as that of non-treated OvCa429 MCAs. The reconstructed map in Fig. 7F shows a heterogeneous distribution of lipids and proteins.

**Figure 8:**
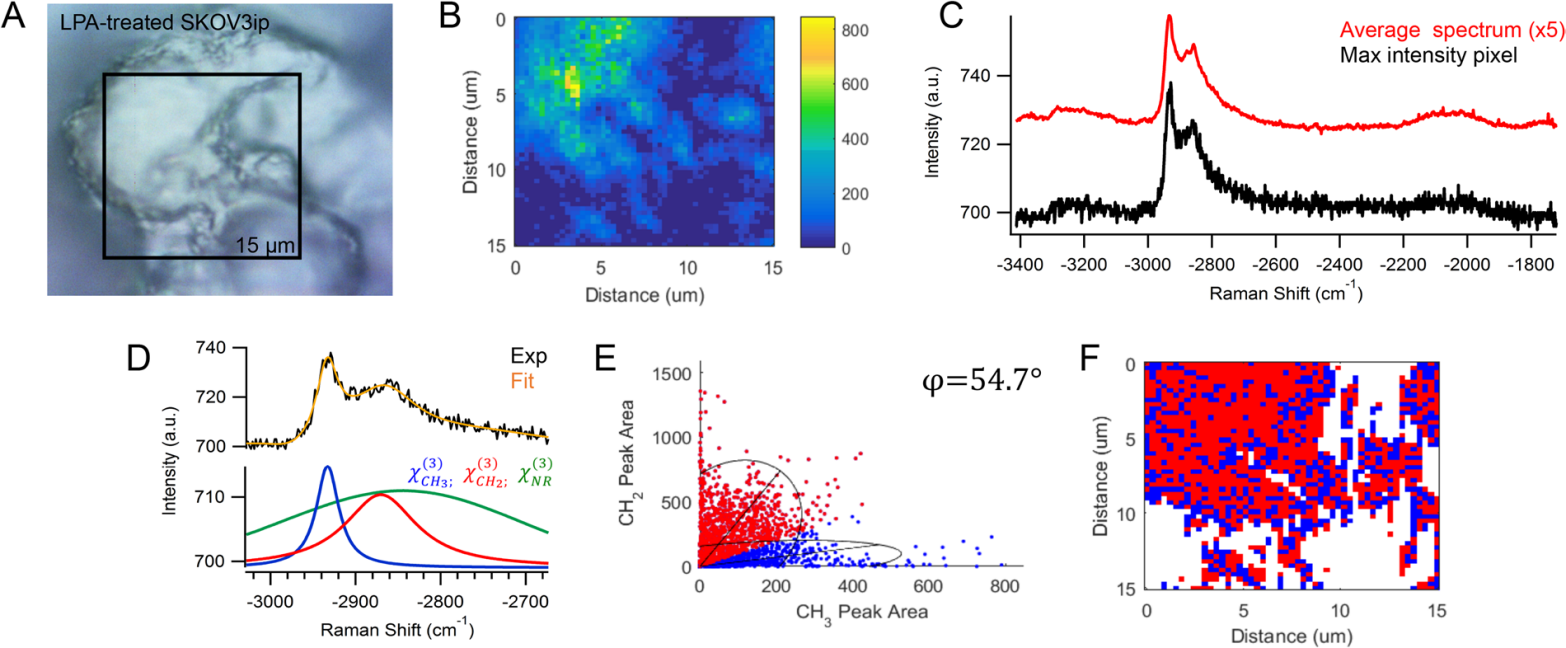
(**A**) Brightfield image of LPA-treated SKOV3ip cells on the surface of the MCA is consistent with (**B**) the CARS image of −2930 cm^−1^ peak area. (**C**) The maximum intensity CARS spectrum and average spectrum of the map show varying contributions of CH_2_ and CH_3_ signal. (**D**) An example of the nonlinear peak fit (orange) of the CARS intensity (black) has contributions from the nonresonant background (green) and resonant vibrational bands at −2850 cm^−1^ (red) and −2930 cm^−1^ (blue). (**E**) The frequency scatter plot exhibits overlapping spectral clusters with 95% confidence ellipses and angle, φ, corresponding to the spectral separation of lipids (red) and proteins (blue). (**F**) The reconstructed map shows the chemical distribution of the segmented lipid (red) and protein (blue) pixels. Experimental parameters: Pump = 720 nm, 25.20 mW; Supercontinuum = 785–945 nm, 3.80 mW; Acquisition time = 500 ms/pixel; Map size = 51 × 51 pixels; Steps = 300 nm; Objective = 100x, 0.9 NA.

After the addition of 80 µM LPA, the SKOV3ip MCAs appear to have a bare cellular membrane with newly formed protrusions in the SEM images in Fig. 6C,D. The CARS image in Fig. 8B correlates well with the brightfield image of LPA-treated SKOV3ip in Fig. 8A. The CARS spectrum of the maximum intensity pixel in Fig. 8C is separated into nonresonant and resonant contributions in Fig. 8D. The peak ratio between CH_2_/CH_3_ functional groups for each spectrum is displayed in Fig. 8E. The chemical distribution map in Fig. 8F exhibits a more uniform coverage of lipids with proteins near the outer perimeter of the cell surface than in the non-treated cells. This distribution can be attributed to the bare lipid membrane of the individual ovarian cancer cells and the protrusions localized between the cell junctions observed in Fig. 6D.

**Figure 9:**
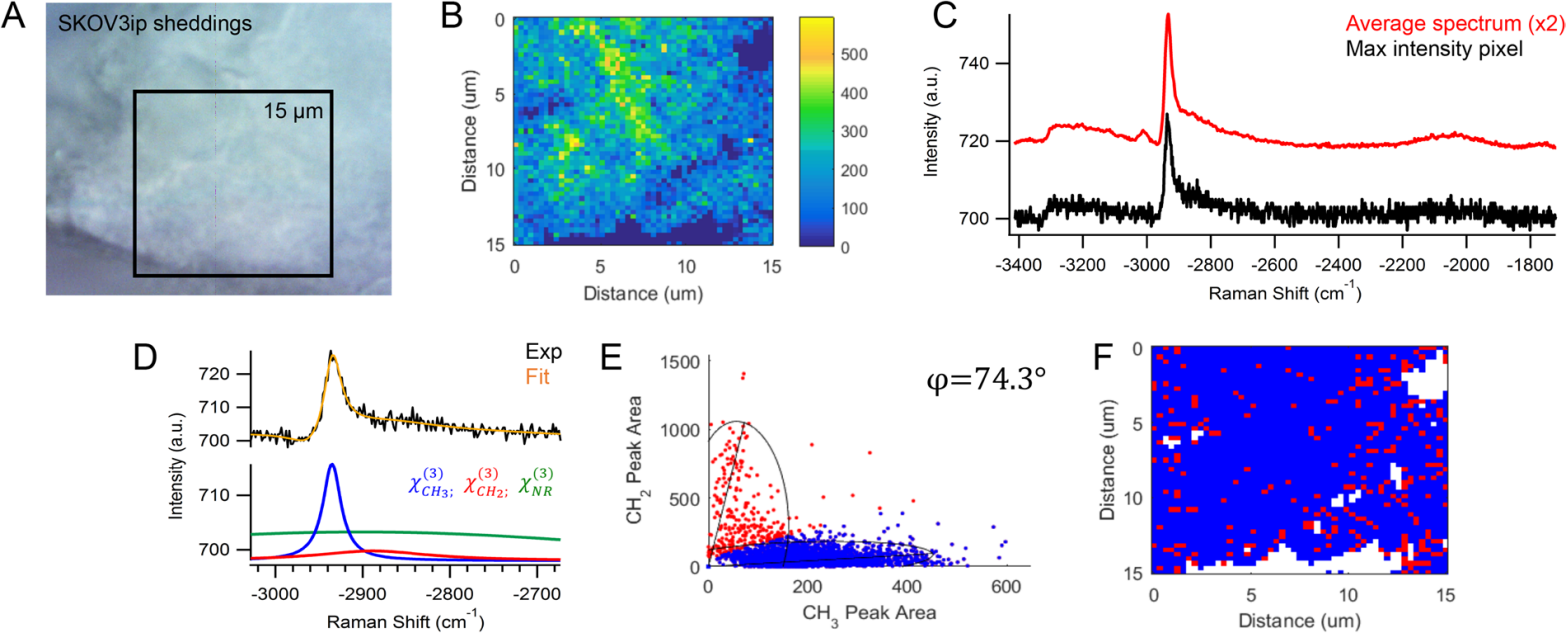
(**A**) Brightfield image of cellular shedding from LPA-treated SKOV3ip has (**B**) corresponding CARS image of −2930 cm^−1^ signal. (**C**) The maximum intensity CARS spectrum and average spectrum of the map show similar chemical composition as non-treated SKOV3ip samples. (**D**) The representative CARS spectrum and nonlinear peak fit results provide the resonant CH_2_ and CH_3_ components used for (**E**) the frequency scatter plot separating the chemical contributions of lipids (red) and proteins (blue). (**F**) The reconstructed map shows the chemical distribution of the segmented lipid (red) and protein (blue) pixels. Experimental parameters: Pump = 720 nm, 25.20 mW; Supercontinuum = 785–945 nm, 3.80 mW; Acquisition time = 500 ms/pixel; Map size = 51 × 51 pixels; Steps = 300 nm; Objective = 100x, 0.9 NA.

The cellular sheddings of the LPA-treated SKOV3ip, depicted in the SEM image in Fig. 6C, were isolated from the conditioned medium and examined. The brightfield and CARS images of the collected sheddings are presented in Fig. 9A and B. The multiplex CARS spectra in Fig. 9C exhibits a decrease in −2850 cm^−1^ that is also observed in Fig. 4C in the OvCa429 sheddings. The frequency scatter plot in Fig. 9E has an identical separation angle to the OvCa429 sheddings in Fig. 4E. The reconstructed map in Fig. 9F shows an abundance of proteins suggesting that the sheddings are comprised of the microvilli and cell-surface proteins originally on the non-treated SKOV3ip surface.

**Figure 10:**
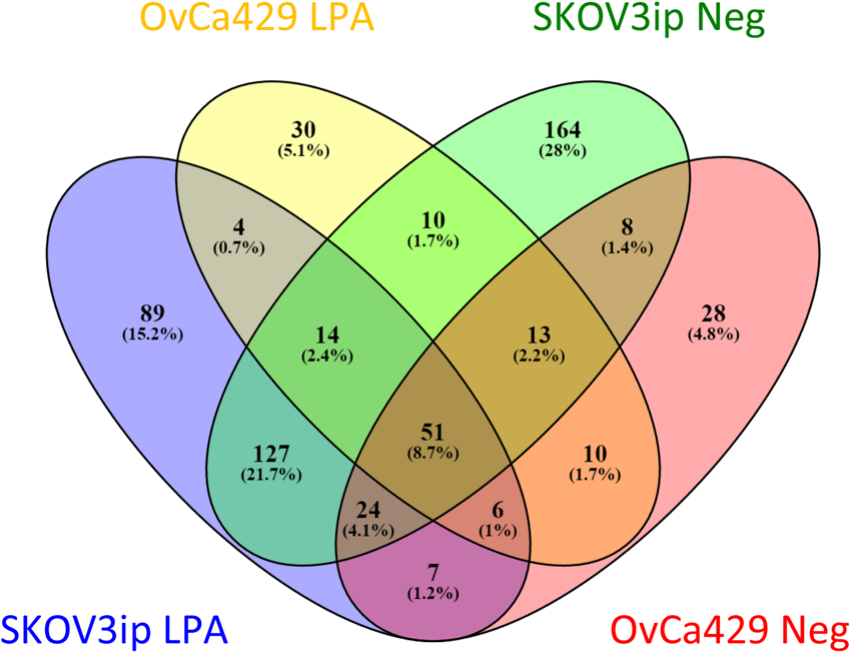
The Venn diagram illustrates the genes associated with the peptide identifications from each cell line with and without LPA treatment.

The sheddings from the LPA-treated OvCa429 and SKOV3ip samples were collected and analyzed using gel electrophoresis and tandem mass spectrometry (MS/MS). From the gel (Fig. S5), more proteins appear to be detected in the SKOV3ip cell line than in the OvCa429 cell line, with or without LPA treatment. The MS/MS results show a range of proteins that could be identified from sequence homology with known human proteins. Examples of base peak chromatograms and the mass spectrum of the most intense peak are shown in supporting information (Fig. S6). Tables of the identified proteins are also provided in the supporting information. Figure 10 shows the Venn diagram of the identified proteins from the LPA-treated sheddings and untreated culture media for both cell lines. In the MS/MS experiments, there are 129 and 142 genes associated with the peptides for LPA-treated and control OvCa429 cells, and for LPA-treated and untreated SKOV3ip cells, 303 and 382 genes, respectively. There are no clear trends in the peptides identified, suggesting that more in depth quantitative experiments are needed to distinguish the specific proteins involved in the LPA-associated morphology changes.

## Discussion

Here we have utilized a suite of analytical techniques to discern the molecular origin of topographical changes on the surface of epithelial ovarian cancer cells in response to LPA treatment. SEM shows pronounced changes in surface structures observed on two different ovarian cancer cell lines. The contrasting peak ratios of methylene, CH_2_, to methyl, CH_3_, functional groups in the CARS signal suggest the relative composition of lipids and proteins are changing. The CARS images provide spatial context to the changes of these biomolecules. Furthermore, the changes in chemical composition of non-treated and LPA-treated MCAs provide a molecular explanation for the observed morphological differences on the cell surface. In Fig. 2F, the uniform distribution of proteins on the cell surface and lipids localized to the outer perimeter of the non-treated OvCa429 sample resemble the physical characteristics of the dense coverage of microvilli in the SEM images in Fig. 1A,B. The chemical distribution map in Fig. 7F has a mixture of lipids and proteins, which is consistent with the irregularly scattered microvilli on the cell surface of non-treated SKOV3ip cells in Fig. 6A,B.

The chemical variations in LPA-treated OvCa429 MCAs and LPA-treated SKOV3ip MCAs in Figs 3F and 8F, correlate with the morphological changes emphasized in the SEM images in Figs 1C,D and 6C,D, respectively. The decrease in protein-associated features is attributed to a loss of microvilli and cell-surface proteins with LPA treatment, since the MCAs appear bare and smooth compared to non-treated MCAs. The multiplex CARS signal from cellular sheddings individually collected from LPA-treated OvCa429 and SKOV3ip MCAs both suggest a high protein composition in Figs 4 and 9. These results support the hypothesis that the cellular sheddings are composed of cleaved microvilli and cell-surface proteins that were originally located on the surface of non-treated OvCa429 and SKOV3ip MCAs.

The CARS images containing several ovarian cancer cells show that cellular aggregates give rise to the same chemical changes observed in individual OvCa429 and SKOV3ip samples. In Fig. 5A,C, the CARS image of non-treated OvCa429 MCAs has a 600 nm step size between each pixel, and the average CARS spectrum exhibits a consistent chemical composition across the surface of the multicellular aggregate. This agrees with the average CARS spectrum from a single ovarian cancer cell in Fig. 2, such that the CARS image has 300 nm steps, equivalent to the lateral resolution of the multiplex CARS microscope.

It is interesting that both the SKOV3ip and OvCa429 show similar morphological changes and decreases in relative surface protein composition with LPA treatment. It has been reported that mesenchymal cell types (e.g. – SKOV3ip) show junction disruption and dispersal in response to LPA^11^, whereas epithelial cell types (e.g. OvCa429) are resistant to this behavior^9,11,43^. Studies have shown specific adherent proteins, such as cadherins^11,13^, which are differentially expressed in the cell types are targeted by LPA treatment. The SEM and CARS images show that both cell types lose microvilli-like structures on the surface. The brightfield images for the LPA treated SKOV3ip cells (Fig. 8A) show evidence of weakened junctions, in agreement with previous reports^43^.

The data presented here, suggest, subject verb agreement with data that LPA treatment alters the magnitude of protein shedding observed between mesenchymal (SKOV3ip) and epithelial (OvCa429) cell types. The relative change with LPA treatment is different between the two cell types. The relative change in the number of pixels being classified as protein or lipid appears greater in the mesenchymal-type cells. Similarly the number of proteins associated with the sheddings in the MS experiments is greater for the mesenchymal-type cells than the epithelial-type cells.

Additional MS experiments are warranted to further explore the effect of LPA treatment. It is known that LPA triggers phosphorylation of many proteins^1^. Phosphorylation is infamously challenging in MS experiments^44^. The lack of obvious trends in the identified proteins suggests the relative expression may be changing, which will require more extensive quantitative MS experiments. It is interesting to note, that some of the proteins detected are associated with exosomes. Cross referencing the detected proteins with the top 100 proteins in the ExoCarta data base^45–47^ shows 25/26 matches for the SKOV3ip cells and 16/19 matches for the OvCa429 cells, with/without LPA treatment.

Taken together, the SEM, CARS, and MS data indicate LPA promotes the shedding of proteins from the surface of ovarian cancer cells, resulting in a morphology change and alterations in the relative protein to lipid ratio. The effect appears to be more pronounced in mesenchymal cells than epithelial cells.

## Conclusion

SEM images show microvilli-like structures are removed from the surface of ovarian cancer cell lines with treatment by LPA. Multiplex CARS imaging, via supercontinuum excitation, provides chemical insight into LPA treated ovarian cancer cells in a label-free, non-destructive manner. From deconvolution of the nonresonant and resonant contributions to the nonlinear CARS intensity in the C-H region between −2800 to −3000 cm^−1^, the peak ratio of methylene to methyl, CH_2_/CH_3_, functional groups can distinguish the relative composition of lipids and proteins in cells. The multiplex CARS approach is necessary to differentiate the CH_2_ and CH_3_ stretches and provide chemical information about biomolecule distributions. Our results indicate that epithelial-type OvCa429 cell line and mesenchymal-type SKOV3ip cell line undergo similar morphological and chemical changes to treatment with LPA. The cellular sheddings collected from LPA-treated cells samples show CARS signals consistent with cleavage of proteins originally present on non-treated cells. MS/MS profiling indicates a number of proteins are found in the culture media with LPA treatment, with the mesenchymal-type SKOV3ip cells shedding a larger number of peptides. Future work quantifying the specific cell-surface proteins and examining the phosphoproteome will provide definitive information regarding which proteins are shed with LPA treatment and the role of this process in ovarian cancer.

## Methods

### Coherent Anti-Stokes Raman Detection

The CARS microscope, illustrated in Fig. S1, consists of an electronically synchronized (Coherent SynchroLock AP, Model 2.0b) picosecond pulsed beam (Coherent Mira Ti:sapphire Model 900-P, 2 ps, 76 MHz) with a broadband femtosecond pulsed beam (Coherent Mira Ti:sapphire Model 900-F, 100 fs, 76 MHz), individually powered by diode pumped solid state lasers (Lighthouse Photonics, Sprout-H, 532 nm, 10 W). The broadband Stokes beam centered at 800 nm is coupled into a 12-cm photonic crystal fiber (NKT Photonics, Femto White 800) to generate a femtosecond pulsed supercontinuum spanning 400 nm to 1100 nm. Both beams are linearly polarized and collinearly combined into an upright microscope equipped with a motorized nosepiece (Scientifica, SliceScope Pro) for focus control. An x-y-z piezo stage (Mad City Labs Inc., Nano-LP200, Nano-Drive) is used for point-by-point imaging.

For all multiplex CARS spectra, the supercontinuum filitered wavelengths between 785–945 nm and pump beam centered at 720 nm give rise to CARS signal from −1800 to −3300 cm^−1^, where the spectral resolution (7–8 cm^−1^) is limited by the grating and size of the charge coupled device (CCD) camera chip and the time-bandwidth product of the pump laser. The pump and supercontinuum laser powers were controlled between 22–27 mW and 3–5 mW, respectively. Two different air objectives were used to excite the individual samples (Olympus MD Plan 0.9 NA and Olympus U Plan FLN 0.75 NA). The CARS signal was measured in the forward-direction with a collection objective (Nikon LPlan 0.70 NA), then focused with another objective (Olympus Plan N 0.25 NA) into an optical fiber and dispersed by a spectrograph onto the CCD camera (Andor iDus).

All mapping parameters were controlled by Labview. The x-y-z piezo stage traveled varying distances of 10–30 µm with step sizes ranging 300–600 nm. The spectral acquisition time of 500 ms/pixel with 1 accumulation resulted in approximately 35 minutes for an average map size of 51 × 51 pixels. Taking into account biological triplicates, 13 maps of non-treated OvCa429, 21 maps of LPA-treated OvCa429, 13 maps of LPA-treated OvCa429 sheddings, and 7 maps of LPA in conditioned medium were recorded, amounting to 54 chemical images for all OvCa429 samples. A total of 24 chemical images were collected for SKOV3ip samples, including 8 maps of non-treated SKOV3ip, 8 maps of LPA-treated SKOV3ip, 6 maps of LPA-treated SKOV3ip sheddings, and 2 maps of LPA in conditioned medium. Data analysis was performed in Matlab (Mathworks) and Igor Pro (Wavemetrics).

### Spontaneous Raman Detection

Spontaneous Raman images were acquired in a commercial micro-Raman spectrometer (Jasco, NRS-5100) with a 532.1 nm laser set at 4.2 mW. An air objective (Olympus MD Plan 0.9 NA) was used for excitation and backscattering collection. Each Raman map is a 15 × 15 µm region composed of 15 × 15 pixels with 5 second/pixel acquisitions, approximately 25 minutes for completion. There were 3 maps of non-treated OvCa429, 4 maps of LPA-treated OvCa429, and 3 maps of LPA-treated OvCa429 sheddings. All data analysis was performed in Matlab (Mathworks) and Igor Pro (Wavemetrics).

### Sample Preparation

#### Cell Line Maintenance and Multicellular Aggregate Formation

The epithelial ovarian carcinoma cell line OvCa429 was provided by Dr. Robert Bast (University of Texas M.D. Anderson Cancer Center, Houston, TX) and maintained in Minimal Essential Medium (MEM; Gibco) containing 10% Fetal Bovine Serum (FBS; Gibco), 1% Non-Essential Amino Acids (NEAA; Corning Cellgro), 1% Penicillin/Streptomycin (Pen/Strep; Lonza), 1% Sodium Pyruvate (Corning Cellgro), 0.1% Amphotericin B (Cellgro). Human epithelial ovarian cancer SKOV3ip cells were obtained from Dr. Katherine Hale (University of Texas M.D. Anderson Cancer Center, Houston, TX) and maintained in RPMI-1640 medium (Corning Cellgro), supplemented with 10% FBS, 1% L-glutamine (Gibco by Life Technologies), 1% sodium pyruvate, 1% Pen/Strep, 1% NEAA, 1% 4-(2-hydroxyethyl)-1-piperazineethanesulfonic acid (Gibco by Life Technologies), and 0.1% Amphotericin B. The LPA-treated cells were maintained similarly with the addition of 80 µM LPA (cat. # 857130 C, Avanti Polar Lipids, Inc.) in the culture medium. The hanging drop method was performed as described previously^48^ with or without addition of 80um LPA into the hanging drops. Briefly, cancer cells were harvested, centrifuged, and re-suspended in fresh medium with and without LPA at 100,000 cells/mL. Droplets (20 µL) were seeded on the inner surface of 150 × 25 mm tissue culture dish lids with approximately 2,000 cells per droplet. Phosphate-buffered saline (PBS) was added to the lower dish and the lid was inverted and placed on the dish. The cells were incubated for an additional 48 hours and non-treated MCAs and LPA-treated MCAs were confirmed under a light microscope.

The cellular sheddings from LPA-treated cell monolayers were collected with the conditioned medium, centrifuged down, and the supernatant was aspirated. The pellet of sheddings was re-suspended in the primary fixative solution (2% Glutaraldehyde, 2% Paraformaldehyde in 0.1 M Cacodylate buffer pH 7.35) and rotated over night at 4 °C. An LPA control sample without ovarian cancer cells was prepared by incubating only LPA and conditioned medium for 24 hours. All samples were prepared in biological triplicates, fixed on poly-L-lysine coated coverslips, and stored in moistened 6-well plates in 4 °C.

#### Sample Preparation for Scanning Electron Microscopy Measurements

Multicellular aggregates of individual untreated and LPA-treated OvCa429 and SKOV3ip samples were collected into 50 ml falcon tubes and centrifuged. The MCA pellets were re-suspended in primary fixative solution (2% Glutaraldehyde, 2% Paraformaldehyde in 0.1 M cacodylate buffer pH 7.35), and rotated for one hour at room temperature in Eppendorf tubes. Washed MCA pellets were evenly distributed in 100 µL Cacodylate buffer and placed on poly-L-lysine coated coverslips and incubated for 30 minutes at room temperature. A microwave vacuum chamber (PELCO® EM Pro Microwave, Ted Pella, Inc.) was used for secondary processing with 2% osmium tetroxide in Cacodylate buffer. The MCAs were washed with ultrapure water three times for five minutes each, dehydrated in series of increasing ethanol concentrations (20%, 50%, 70% 90%, and 3 × 100%), then critical point dried using a Autosamdri-931 critical point dryer (Tousimis Research Corporation). All samples were placed on individual carbon stubs, sputter coated with iridium and examined under an extreme high resolution scanning electron microscope (FEI-Magellan 400).

#### Sample Preparation for Proteomic Profiling

Cell shedding collections and negative control conditioned media samples were dissolved in 20 uL of 8 M urea/2 M thiourea buffer in 100 mM ammonium bicarbonate (pH 8) and incubated at 37 °C for 1 hour. The samples were then centrifuged at 2000g to pellet undissolved material and the supernatant was transferred to a separate tube. Samples (10 μL volume) were reduced with 10 mM dithiothreitol at 60 °C, then alkylated with 25 mM iodoacetamide at room temperature in the dark. A 5 μL volume of 2% SDS was mixed into the samples, and then 5 μL gel loading buffer was added and the solutions vortexed.

A commercial bis-Tris mini-SDS-PAGE gel (4–12%, NuPAGE) was loaded with sample (30 µL per well, one sample per lane). The gel was run for 1 hour on standard ‘NuPAGE’ settings (200 volts, 120 milliamps, 25 watt maximums). After electrophoresis, the gel was fixed and stained overnight with Coomassie Colloidal Brilliant Blue staining kit. The gel was destained with distilled water for 3 hours until the background was clear. The gel was divided into six bands of roughly equal protein amount for each sample lane, and in-gel digestion proceeded according to Shevchenko *et al*.^49^. After digestion, the six fractions per lane were combined into three fractions (1 and 2, 3 and 4, and 5 and 6). Samples were desalted using C18 Ziptips (Millipore) and resuspended in 10 µL 0.1% formic acid in MS grade water.

#### UPLC/nESI-MS/MS Analysis

A Waters nanoACQUITY Ultra-performance LC system (Milford, MA, USA) was used for separation of gel fractions. Buffer A (0.1% formic acid in water) and Buffer B (0.1% formic acid in acetonitrile) were used as the mobile phases for the separation. For all the runs, 1 μL (ca. 1 μg) of peptides were loaded onto a C18 reverse-phase column (Waters, 100 μm × 100 mm, 1.7 µm particle size, BEH130C18) held at 40 °C. Samples were run with 2% buffer B for 12 minutes at 1 µL/min, followed by 60 minutes from 8% buffer B to 30% Buffer B at 0.7 μL/min. The column was washed at 80% buffer B at 1 µL/min, followed by equilibration for 12 minutes at 2% buffer B at 1 µL/min. The total UPLC method time was 90 minutes. The eluted peptides were pumped through a nano-ESI spray emitter and analyzed by the Q-Exactive instrument (Thermo Fisher Scientific, Waltham, MA, USA). The electrospray voltage was 1.8 kV. Each fraction was run in technical duplicate. A top 20 data dependent acquisition (DDA) method was used. Full MS scans were acquired in the Orbitrap mass analyzer over m/z 350–1800 range with resolution 70,000 (m/z 400). The target value was 1.00E + 06. Twenty most intense peaks with charge state ≥2 were selected for sequencing and fragmented in the ion trap with normalized collision energy of 30, isolation window of 2 m/z units and one microscan. Dynamic exclusion was enabled, and peaks selected for fragmentation more than once within 30 seconds were excluded from selection for 60 seconds.

Data analysis was performed using Proteome Discoverer (ver.1.4). Data was searched with MASCOT against the Uniprot human database (version: 2.5, Number of sequences: 19,210) using a precursor mass tolerance of 20 ppm and fragment mass tolerance of 0.5 Da. The search included enzyme as trypsin, variable modifications of methionine oxidation, protein N-terminal and lysine acetylation and deamidation (NQ), and fixed modification of carbamidomethyl cysteine. The maximum number of missed cleavages was set to two. A Top N filter was set to 12 per 100 Da mass range. The Percolator node was added for PSM validation. Data was filtered by peptide confidence set to high. Data was reduced by removing all identifications resulting from single PSMs.

### Availability of data and material

The data generated or analyzed during this study are included in this published article and its supplementary information files. Raw data files for CARS images are available from the corresponding author on reasonable request.

## Electronic supplementary material

Supporting Information

MS Dataset
