# Supplementary material for: Chemical Analysis of Morphological Changes in Lysophosphatidic Acid-Treated Ovarian Cancer Cells
Source: Sci Rep. 2017 Nov 10;7:15295. doi: 10.1038/s41598-017-15547-7 (PMC5681516; doi:10.1038/s41598-017-15547-7)
Supplement: Supplementary file 1 — Supporting Information [file 41598_2017_15547_MOESM1_ESM.pdf]

SUPPORTING INFORMATION: CHEMICAL ANALYSIS OF MORPHOLOGICAL CHANGES IN LYSOPHOSPHATIDIC ACID-TREATED OVARIAN CANCER CELLS

Karen A. Bailey, Yuliya Klymenko, Peter Feist, Amanda B. Hummon, M. Sharon Stack, and Zachary D. Schultz

University of Notre Dame, Department of Chemistry and Biochemistry, Notre Dame, IN 46556.

Harper Cancer Research Institute, University of Notre Dame, Notre Dame, IN 46556

Contents:

Figure S-1 .... S-2

Figure S-2 .... S-3

Figure S-3 .... S-5

Figure S-4 .... S-6

Figure S-5 .... S-7

Figure S-6 .... S-8

MatLab Script for Nonlinear Peak Fits S-9

Table S-1 .... S-20

MS identifications .... xlsx file

Figure S-1

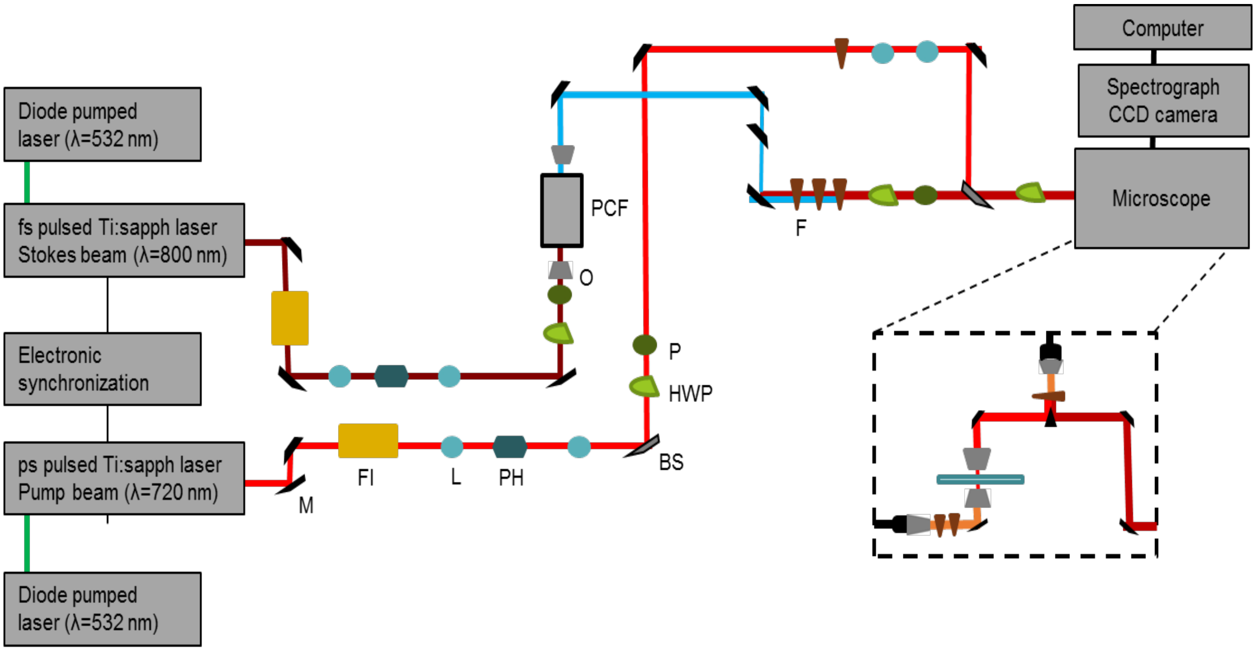

Figure S-1. Schematic of the CARS microscope consists of an electronically synchronized picosecond pulsed beam with a broadband femtosecond pulsed beam, individually powered by diode pumped solid state lasers. There Stokes beam path generates a supercontinuum excitation by focusing the Stokes beam through an objective (O) onto a photonic crystal fiber (PCF). Both beams are linearly polarized and collinearly combined into an upright microscope equipped with a motorized nosepiece for focus control. An x-y-z piezo stage is used for point-by-point imaging. Optical path includes: M=mirror, FI=Faraday isolator, L=lens, PH=pinhole, BS=beam splitter, HWP=half wave plate, P=polarizer, and F=filter.

Figure S-2

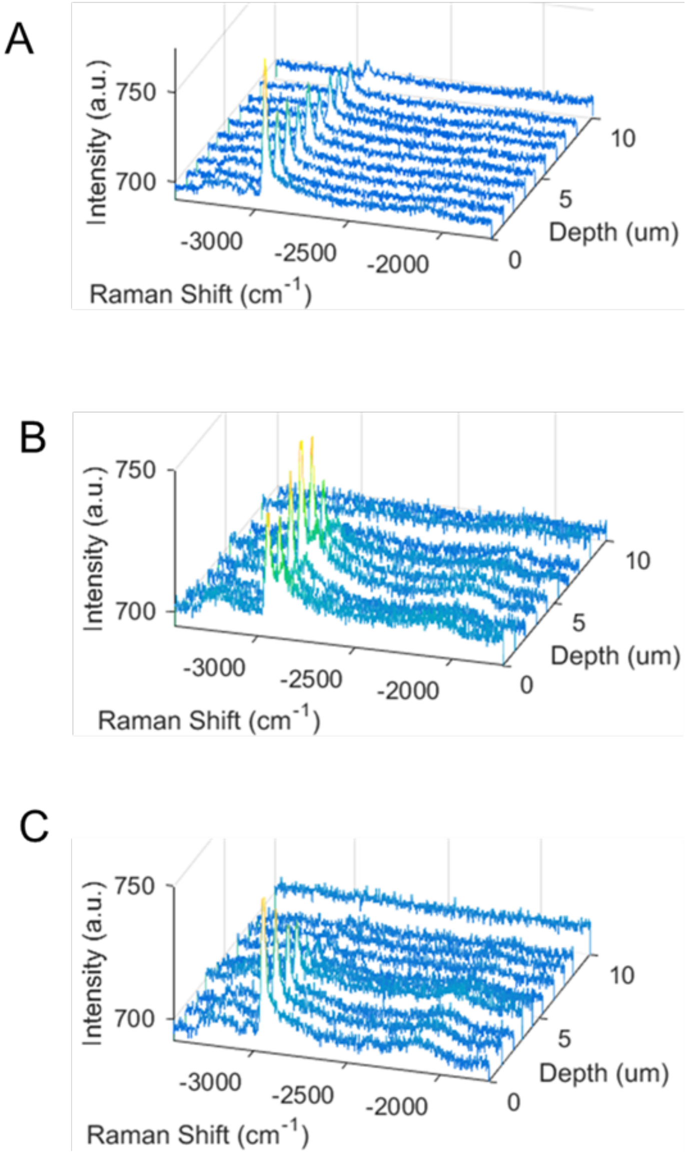

Figure S-2. Waterfall plots as a function of depth for the CARS spectra for (A) non-treated OvCa429 cells, (B) LPA-treated OvCa429 and (C) OvCa429 cellular shedding measured at varying sample depths.

The waterfall plots in Figure S-2 are multiplex CARS spectra collected from non-treated OvCa429 MCAs, LPA-treated OvCa429 MCAs, and cellular sheddings from LPA-treated OvCa429 MCAs, respectively. Although the CARS intensity decreases as the depth increases, the peak ratio between the different vibrational bands is comparable to the measured CARS signal in Figures 2-4 of the individual OvCa429 samples. The decrease in intensity arises from the 4-pi nature of our collection. The focus was moved into the sample; however, the collection lens remained fix; thus accurately measuring the focal volume investigated. The majority of the CARS signal from each sample in Figure S-2 is detected within 5 microns from the cell surface of the multicellular aggregates. This is consistent with the axial resolution of our multiplex CARS instrument, such that the coherence length is approximately 2-3 microns, corresponding to the estimated full width at half maximum of the CARS intensity in relation to the axial distance.

Figure S-3

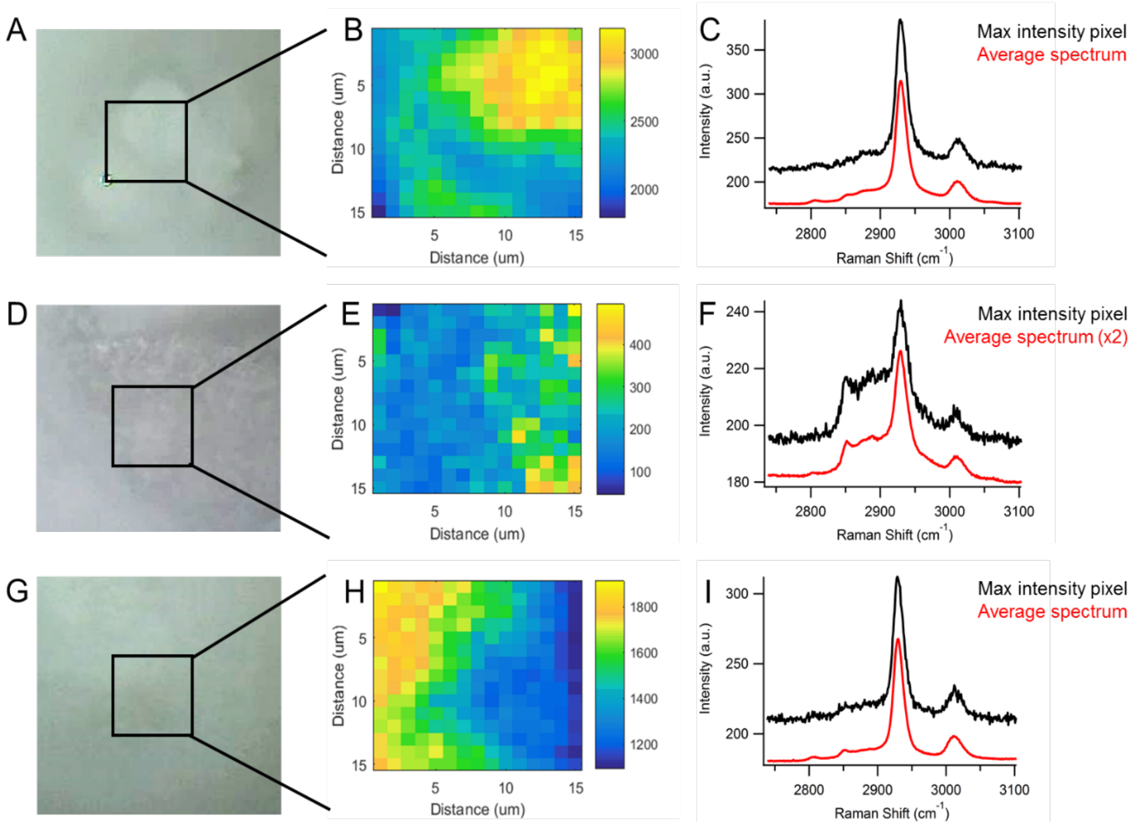

Figure S-3. Brightfield images of (A) non-treated OvCa429 cells, (D) LPA-treated OvCa429 cells and (G) OvCa429 cellular shedding have (B, E, H) corresponding Raman images of - 2930 cm^{-1} signal. (C, F, I) The vibrational bands in the maximum intensity Raman spectrum and average spectrum of each sample are in good agreement with CARS measurements. Experimental parameters: Laser=532.1 nm, 4.2 mW; Acquisition time=5 seconds/pixel; Map size=25x25 pixels; Steps=1 μm; Objective=100x, 0.9 NA.

Figure S-4

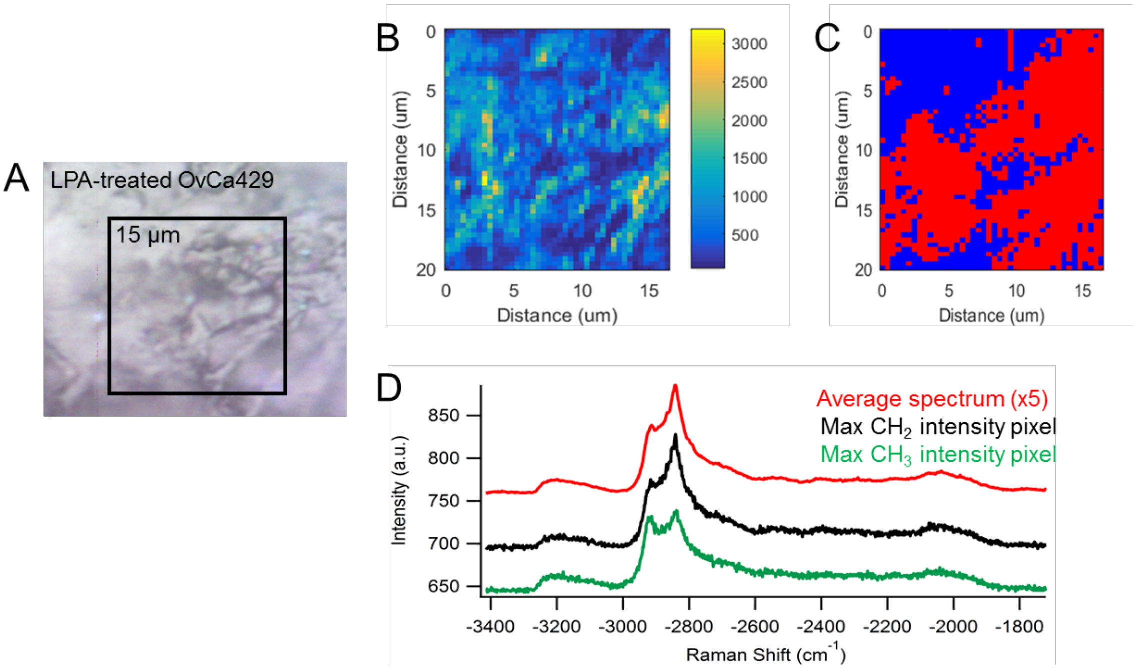

Figure S-4. The bright features in the (A) brightfield image of OvCa429 cells is characteristic of crystalline LPA on the cell surface. (B) The CARS image of -2850 cm^{-1} signal resembles the morphological features in (A). (C) The reconstructed map of lipid (red) and protein (blue) pixels illustrate the distribution of high lipid signal indicative of LPA. Representative CARS spectra of the maximum CH_3 intensity pixel, maximum CH_2 intensity pixel, and map average are shown in (D). Experimental parameters: Pump=720 nm, 25.50 mW; Supercontinuum=785-945 nm, 4.60 mW; Acquisition time=500 ms/pixel; Map size=51x42 pixels; Steps=400 nm; Objective = 100x, 0.9 NA.

Figure S-5

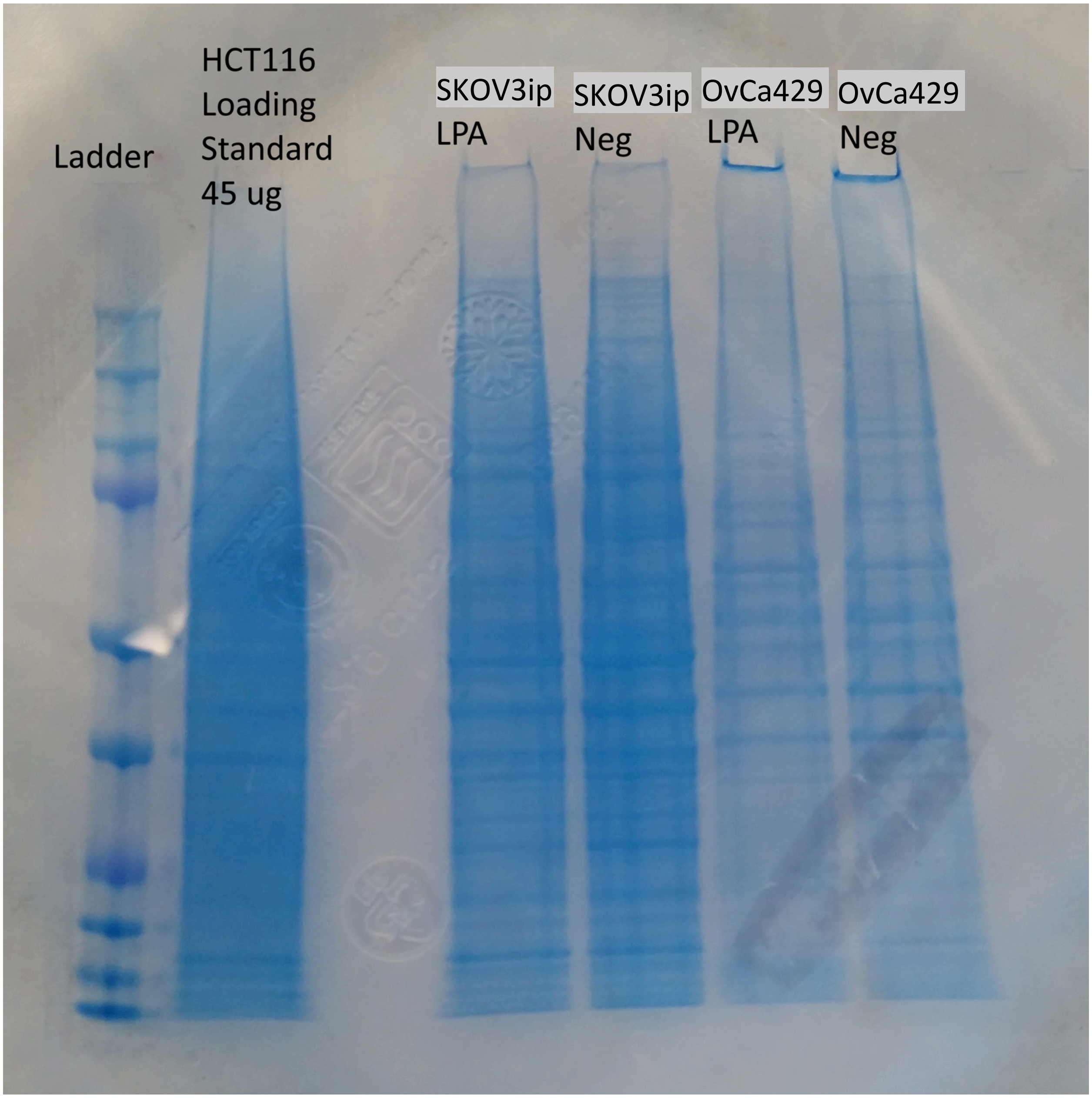

Figure S-5. The photograph shows the gel and the bands observed from the media environment associated with sheddings for both OvCa429 and SKOV3ip cell lines. Similarities are observed between the LPA treated and negative controls for each cell line. The two cell lines show differences between them. Both show fewer signals than the HCT116 loading standard.

Figure S-6

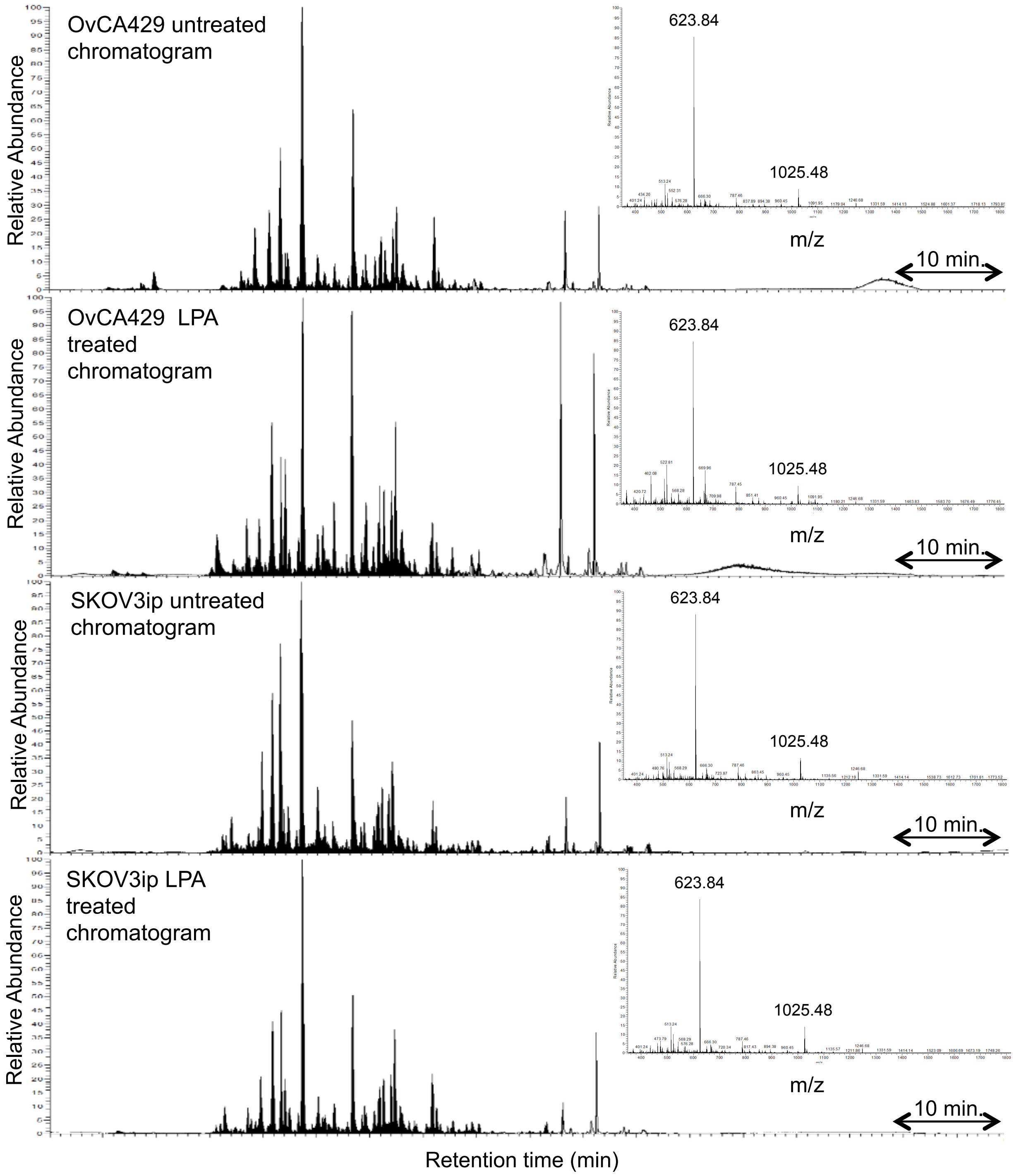

Figure S-6. Base peak chromatograms for the first gel fraction of each sample are shown. The insets show the mass spectrum of the most intense peak in the chromatogram.

Matlab Script for Nonlinear Peak Fits and Scatter Plots

1. Nonlinear Equations for Script

The CARS signal, I_{CARS}, can be written as:

$$I_{CARS} \propto \left| \chi_{NR}^{(3)} + \frac{A_{R1}}{(\omega_P - \omega_S - \Omega_{R1}) - i\Gamma_1} + \frac{A_{R2}}{(\omega_P - \omega_S - \Omega_{R2}) - i\Gamma_{R2}} \right|^2 \quad \text{(Eq. S-1)}$$

where the nonresonant term, $\chi_{NR}^{(3)}$, is:

$$\chi_{NR}^{(3)} = A_{NR} e^{it - \left(\frac{(\omega_P - \omega_S - \omega_{NR})^2}{2\Gamma_{NR}^2}\right)} \quad \text{(Eq. S-2)}$$

All fit functions were created in Curve Fitting Tool (cftool) which generated individual Matlab code files. The coefficients in the script are assigned in Table S-1. The first term in Eq. S-2 corresponds to the nonresonant background to account for any coherent interference and contributions from the supercontinuum excitation beam and surrounding medium. The second and third terms in Eq. S-1 refer to the resonant contributions of CH_3 and CH_2 vibrational bands, respectively.

1.1 Example Matrices in Workspace

```
%% Import data set from file and separate map position and signal intensities
ovcasample1map1=dlmread('sample1-map1-15x15um-51seps-500ms-1acc-0.9naobj');
ovcasample1map1=ovcasample1map1(:,3:1026);
%% Isolate spectral region of interest
ovcasample1map1=ovcasample1map1(:,201:400);
%% Remove dark counts and normalize by subtracting the minimum intensity
```

```
for k=1:2601; ovcasample1map1s(k,:)=ovcasample1map1(k,:)-
min(ovcasample1map1(k,:);end
%% Import reference and generate wavenumber region of interest
ovcasample1ref=dlmread(‘sample1-reference.asc’)
wavenumber=ovcasample1ref(:,1);
wavenumberfit=wavenumber(201:400,:);
%%Create a sample spectrum from map for peak fit script
ovcasample1map1spectrum=ovcasample1map1(1,:);
%% Create an index vector the same length as spectral region of interest
x=1:200;
```

2. Peak Fit Functions

The following functions only vary in coefficient parameters based on signal intensity of the resonant terms.

2.1. Function 1

%% Function “peakfit_customlorgaus_pch3” is for spectra with high CH_3 signal

```
function [fitresult, gof] = peakfit_customlorgaus_pch3(x, skovsample3rep1map3sp11)
%% Fit: 'CH3 Peak'
 [xData, yData] = prepareCurveData( x, ovcasample1map1spectrum);
% Set up fittype and options.
ft = fittype( 'abs((A1*exp((1i*t)-((x-c1)^2)/(2*(w1^2))))+((A2/((x-c2)-(1i*w2)))+(A3/((x-
c3)-(1i*w3)))))^2)', 'independent', 'x', 'dependent', 'y' );
opts = fitoptions( 'Method', 'NonlinearLeastSquares' );
```

```
opts.Display = 'Off';

opts.Lower = [-Inf 0 0 70 0 0 -Inf 0 0 0];

opts.StartPoint = [-5 15 5 80 50 100 0.965 100 15 20];

% Fit model to data.

[fitresult, gof] = fit( xData, yData, ft, opts );

% Plot fit with data and label axes

figure( 'Name', 'CH3 Peak' );

h = plot( fitresult, xData, yData );

legend( h,  ovcasample1map1spectrum vs. x', 'CH3 Peak', 'Location', 'NorthEast' );

xlabel index

ylabel intensity

grid on
```

2.2. Function 2

```
%% Function "peakfit_customlorgaus_pch2" is for spectra with high CH2 signal

function [fitresult, gof] = peakfit_customlorgaus_pch2(x, ovcasample2map1spectrum)

%% Fit: 'CH2 Peak'.

[xData, yData] = prepareCurveData( x, ovcasample2map1spectrum );

% Set up fittype and options.

ft = fittype( 'abs((A1*exp((1i*t)-((x-c1)^2)/(2*(w1^2))))+((A2/((x-c2)-(1i*w2)))+(A3/((x-
c3)-(1i*w3)))))^2)', 'independent', 'x', 'dependent', 'y' );

opts = fitoptions( 'Method', 'NonlinearLeastSquares' );

opts.Display = 'Off';
```

```
opts.StartPoint = [-10 30 25 80 50 100 0.9449 50 10 30];
% Fit model to data.
[fitresult, gof] = fit( xData, yData, ft, opts );
    % Plot fit with data and label axes
figure( 'Name', ' CH2 Peak'' );
h = plot( fitresult, xData, yData );
legend( h, ' ovcasample2map1spectrum vs. x', ' CH2 Peak', 'Location', 'NorthEast' );
xlabel index
ylabel intensity
grid on
```

2.3. Function 3

```
%% Function "peakfit_customlorgaus_lowsignal" is adjusted for low CARS signal
function [fitresult, gof] = peakfit_customlorgaus_lowsignal(x, ovcasample1map1spectrum)
%% Fit: 'Low Signal'.
[xData, yData] = prepareCurveData( x, ovcasample1map1spectrum );
% Set up fittype and options.
ft = fittype( 'abs((A1*exp((1i*t)-((x-c1)^2)/(2*(w1^2))))+((A2/((x-c2)-(1i*w2)))+(A3/((x-
c3)-(1i*w3)))))^2)', 'independent', 'x', 'dependent', 'y' );
opts = fitoptions( 'Method', 'NonlinearLeastSquares' );
opts.Display = 'Off';
opts.StartPoint = [-2 8 6 75 50 100 0.036329 50 5 30];
% Fit model to data.
```

```
[fitresult, gof] = fit( xData, yData, ft, opts );
% Plot fit with data and label axes
figure( 'Name', 'Low Signal' );
h = plot( fitresult, xData, yData );
legend( h, ' ovcasample1map1spectrum vs. x', 'Low Signal', 'Location', 'NorthEast' );
xlabel index
ylabel intensity
grid on
```

2.4. Function 4

```
%% Function "peakfit_customlorgaus_noise" is for spectra without CARS signal which fits
the nonresonant background to the baseline
function [fitresult, gof] = peakfit_customlorgaus_noise(x, ovcasample1map1spectrum)
%% Fit: 'Noise'.
[xData, yData] = prepareCurveData( x, skovsample2rep1map4s1l42 );
% Set up fittype and options.
ft = fittype( 'abs((A1*exp((1i*t)-((x-c1)^2)/(2*(w1^2))))+((A2/((x-c2)-(1i*w2)))+(A3/((x-
c3)-(1i*w3)))))^2)', 'independent', 'x', 'dependent', 'y' );
opts = fitoptions( 'Method', 'NonlinearLeastSquares' );
opts.Display = 'Off';
opts.Lower = [0 0 0 0 0 0 -Inf 0 0 0];
opts.StartPoint = [1 0 0 80 0 0 0.004634 100 0 0];
opts.Upper = [Inf 0 0 Inf 0 Inf Inf Inf 0 0];
```

```
% Fit model to data.

[fitresult, gof] = fit( xData, yData, ft, opts );

% Plot fit with data and label axes

figure( 'Name', 'Noise');

h = plot( fitresult, xData, yData );

legend( h, ' ovcasample1map1spectrum vs. x', 'Noise', 'Location', 'NorthEast' );

xlabel index

ylabel

grid on
```

3. For-Loop Fitting Script

This script is designed to fit consecutive spectra, such that each fit will produce a figure, where k denotes the index position of the spectrum that is analyzed in the map. All fitted lines and coefficients are simultaneously exported to individual matrices in the workspace, which will change dimensions based on k value.

```
%% Change peak fit function to best fit each spectrum

for k=1:50;

spectrum=ovcasample1map1s(k,:); %% Sample maps have 2601 spectra

p=peakfit_customlorgaus_pch3(x,spectrum);

ovcasample1map1fitline (:,k)=p(x);

ovcasample1map1coeff(k,:)=coeffvalues(p);

end
```

A.1 Reconstruction of Resonant and Nonresonant Terms

```
%% Isolate each coefficient from fitting results
ovcasample1map1coeffA1=(ovcasample1map1coeff(:,1));
ovcasample1map1coeffA2=(ovcasample1map1coeff(:,2));
ovcasample1map1coeffA3=(ovcasample1map1coeff(:,3));
ovcasample1map1coeffc1=(ovcasample1map1coeff(:,4));
ovcasample1map1coeffc2=(ovcasample1map1coeff(:,5));
ovcasample1map1coeffc3=(ovcasample1map1coeff(:,6));
ovcasample1map1coefft=(ovcasample1map1coeff(:,7));
ovcasample1map1coeffw1=(ovcasample1map1coeff(:,8));
ovcasample1map1coeffw2=(ovcasample1map1coeff(:,9));
ovcasample1map1coeffw3=(ovcasample1map1coeff(:,10));
%% Reconstruct resonant CH3 contribution as a Lorentzian peak
for k=1:200; for m=1:2601;
ovcasample1map1pch3(m,k)=abs((ovcasample1map1coeffA2(m,:)/((x(:,k)-
ovcasample1map1coeffc2(m,:)-(1i*ovcasample1map1coeffw2(m,:))))))^2);end;end
%% Reconstruct resonant CH2 contribution as a Lorentzian peak
for k=1:200; for m=1:2601;
ovcasample1map1pch2(m,k)=abs((ovcasample1map1coeffA3(m,:)/((x(:,k)-
ovcasample1map1coeffc3(m,:)-(1i*ovcasample1map1coeffw3(m,:))))))^2);end;end
%% Reconstruct nonresonant background as a Lorentzian-Gaussian peak
for k=1:200; for m=1:2601;
ovcasample1map1pnrb(m,k)=abs(ovcasample1map1coeffA1(m,:)*exp((1i*ovcasample1m
```

```
ap1coefft(m,:)-((x(:,k)-
ovcasample1map1coeffc1(m,:))^2)/(2*(ovcasample1map1coeffw1(m,:)^2)))));end;end
%% Calculate the peak area for each resonant contribution
for k=1:2601; ovcasample1map1pch3trapz(k,:)=trapz(ovcasample1map1pch3(k,:));end
for k=1:2601; ovcasample1map1pch2trapz(k,:)=trapz(ovcasample1map1pch2(k,:));end
%% Generate CARS maps
ovcasample1map1pch3trapzc=reshape(ovcasample1map1pch3trapz,51,51,1);
ovcasample1map1pch2trapzc=reshape(ovcasample1map1pch2trapz,51,51,1);
distance=1:1:15;
imagesc(distance, distance, ovcsample1map1pch3trapzc)
%% Plot fitting example with offset adjustment from previous normalization and repeat for
all or any desired terms
for k=1:2601;
ovcasample1map1pch3overlayspectra(k,:)=ovcasample1map1pch3(k,:)+min(ovcasample1
map1s(k,:);
plot(wavenumberfit, ovcasample1map1pch3overlayspectra(1,:))
```

4. Scatter Plot Analysis

4.1. Scatter Groups

```
%% Create a scatter plot
scatter(ovcasample1map1pch3trapz, ovcasample1map1pch2trapz)
%% Create scatter groups, where m and n parameters are adjusted based on distribution
from scatter plot
```

```
scattergroup=[ovcasample1map1pch3trapz ovcasample1map1pch2trapz]
m=find(ovcasample1map1pch3trapz>ovcasample1map1pch2trapz);
ovcasample1map1pch3group=scattergroup(m,:);
n=find(ovcasample1map1pch3trapz<ovcasample1map1pch2trapz);
ovcasample1map1pch2group=scattergroup(n,:);
```

4.2. Error Ellipse

The following code was provided by Mathworks Community – File Exchange and customized for this data set. After reconstructing the error ellipse in the workspace, the major axis line is generated in cftool. The slope of the major axis for each CH_3 and CH_2 scatter group is used to calculate the separation angle between the two contributions.

```
%% Specify scatter group
data = [ovcasample1map1pch3ngroup(:,1) ovcasample1map1pch3ngroup(:,2)];
% Calculate the eigenvectors and eigenvalues
covariance = cov(data);
[eigenvec, eigenval ] = eig(covariance);
% Get the index of the largest eigenvector
[largest_eigenvec_ind_c, r] = find(eigenval == max(max(eigenval)));
largest_eigenvec = eigenvec(:, largest_eigenvec_ind_c);
% Get the largest eigenvalue
largest_eigenval = max(max(eigenval));
% Get the smallest eigenvector and eigenvalue
if(largest_eigenvec_ind_c == 1)
smallest_eigenval = max(eigenval(:,2))
```

```
smallest_eigenvec = eigenvec(:,2);
        else
                smallest_eigenval = max(eigenval(:,1))
                smallest_eigenvec = eigenvec(1,:);
        end
% Calculate the angle between the x-axis and the largest eigenvector
angle = atan2(largest_eigenvec(2), largest_eigenvec(1));
% Shift the angle such that the angle is between 0 and 2pi
if(angle < 0)
        angle = angle + 2*pi;
end
% Get the coordinates of the data mean
avg = mean(data);
% Get the 95% confidence interval error ellipse
chisquare_val = 2.4477;
theta_grid = linspace(0,2*pi);
phi = angle;
X0=avg(1);
Y0=avg(2);
a=chisquare_val*sqrt(largest_eigenval);
b=chisquare_val*sqrt(smallest_eigenval);
% The ellipse in x and y coordinates
ellipse_x_r  = a*cos( theta_grid );
```

```
ellipse_y_r  = b*sin( theta_grid );

% Define a rotation matrix

R = [ cos(phi) sin(phi); -sin(phi) cos(phi) ];

% Rotate the ellipse to some angle phi

r_ellipse = [ellipse_x_r;ellipse_y_r]' * R;

% Draw the error ellipse

plot(r_ellipse(:,1) + X0,r_ellipse(:,2) + Y0,'-')

hold on;

% Plot the original data

plot(data(:,1), data(:,2), '.');

mindata = min(min(data));

maxdata = max(max(data));

xlim([mindata-3, maxdata+3]);

ylim([mindata-3, maxdata+3]);

hold on;

% Plot the eigenvectors

quiver(X0, Y0, largest_eigenvec(1)*sqrt(largest_eigenval),

largest_eigenvec(2)*sqrt(largest_eigenval), '-m', 'LineWidth',2);

quiver(X0, Y0, smallest_eigenvec(1)*sqrt(smallest_eigenval),

smallest_eigenvec(2)*sqrt(smallest_eigenval), '-g', 'LineWidth',2);

hold on;

% Set the axis labels

hXLabel = xlabel('CH_3 Peak Area');
```

```
hYLabel = ylabel('CH_2 Peak Area');

%% Reconstruct ellipse in workspace

ovcasample1map1pch3ellipse=[(r_ellipse(:,1) + X0) (r_ellipse(:,2) + Y0)];

%% Calculate the separation angle between two scatter groups

ovcasample1map1separationdegree=rad2deg(atan(ovcasample1map1pch3slope)-
atan(ovcasample1map1pch2slope));
```

TABLE S-1. Coefficient notation for peak fitting equations

Peak Fitting Notation	Terms in Eq. A.1 and Eq. A.2	Description
A_1	A_{NR}	Amplitude of nonresonant background
A_2	A_{R1}	Amplitude of resonant peak at -2930 cm^{-1}
A_3	A_{R2}	Amplitude of resonant peak at -2850 cm^{-1}
c_1	ω_{NR}	Peak center of nonresonant background
c_2	Ω_{R1}	Center of resonant peak at -2930 cm^{-1}
c_3	Ω_{R2}	Center of resonant peak at -2850 cm^{-1}
t	t	Divergence angle
w_1	Γ_{NR}	Line width of nonresonant background
w_2	Γ_{R1}	Line width of resonant peak at -2930 cm^{-1}
w_3	Γ_{R2}	Line width of resonant peak at -2850 cm^{-1}
x	$\omega_P - \omega_S$	Beat frequency, Raman shift along x-axis
y	I_{CARS}	Signal intensity
